# Dietary Exposure to United States Food and Drug Administration-Approved Synthetic Food Colors in Children, Pregnant Women, and Women of Childbearing Age Living in the United States

**DOI:** 10.3390/ijerph19159661

**Published:** 2022-08-05

**Authors:** Asa Bradman, Rosemary Castorina, Ruwan Thilakaratne, Mayela Gillan, Teja Pattabhiraman, Anuroop Nirula, Melanie Marty, Mark D. Miller

**Affiliations:** 1 Center for Environmental Research and Community Health (CERCH), School of Public Health, University of California at Berkeley, 1995 University Avenue, Suite 265, Berkeley, CA 94704, USA; 2 Department of Public Health, School of Social Sciences, Humanities, and Art, University of California at Merced, 5200 N. Lake Road, Merced, CA 95343, USA; 3 California Office of Environmental Health Hazard Assessment, 1515 Clay Street, 16th Floor, Oakland, CA 94612, USA

**Keywords:** human exposure, food dye, erythrosine, children, pregnant women, ADI, NHANES

## Abstract

The Food and Drug Administration (FDA) regulates artificial food colors (AFCs) in the United States. Exposure to AFCs has raised concerns about adverse behavioral effects in children. We quantified AFC exposure in women of childbearing age, pregnant women, and children and compared them to FDA and World Health Organization acceptable daily intakes (ADIs). We estimated the “typical” and “high” single-day and two-day average dietary exposure to each AFC (mg/kg/day) based on laboratory measurements and food consumption data from the 2015–2016 National Health and Nutrition Examination Survey (NHANES). We also examined whether AFC intake differed by income, education, and ethnicity. Exposure tended to be higher in children and the highest AFC exposure was found for Red No. 40. Children’s mean and 95th percentile FD&C Red No. 3 estimated intakes exceeded the ADIs in some instances. Juice drinks, soft drinks, icings, and ice cream cones were major foods contributing to children’s (<16 years old) AFC exposure. AFC intake was higher in participants with lower incomes and education and of African American ethnicity. The findings indicate widespread AFC exposure including in very young children. Research is needed on the sociodemographic determinants of exposure and AFC toxicokinetics to better describe the absorption and organ-specific exposure.

## 1. Introduction

Color additives of both natural and synthetic origin are commonly added to food, vitamins, drugs, and cosmetic products in the United States (U.S.). Certified color additives are synthetic colors that provide intense, uniform color and can be blended to create a variety of hues. Governmental oversight of the production and use of color additives in the U.S. began in 1938 with the Federal Food, Drug, and Cosmetic (FD&C) Act and continues today. In the U.S., the Food and Drug Administration (FDA) has regulatory oversight of color additives. These artificial food colors (AFCs) are required to be certified for identity and purity every time a new batch is manufactured [1]. The FDA oversees the batch certification of color additives and monitors their use in consumer products including the labeling of products containing AFCs [2].

Currently, there are seven FD&C color additives approved for general use in food in the United States: FD&C Blue No. 1 (Brilliant Blue), FD&C Blue No. 2 (Indigo Carmine), FD&C Green No. 3 (Fast Green), FD&C Red No. 3 (Erythrosine), FD&C Red No. 40 (Allura Red), FD&C Yellow No. 5 (Tartrazine), and FD&C Yellow No. 6 (Sunset Yellow). These AFCs are used to increase the visual appeal of foods, simplify the identification of the various flavors of a product, and to even out naturally occurring color variations [3]. These dyes are known as “straights” or color additives that have not undergone chemical reactions with other substances. Additives that are reacted with alumina hydrate metallic salts/precipitants and substrata to create powders are known as “lakes” and are often used in bakery products [4,5].

Concerns have been raised about the adverse neurodevelopmental or behavioral effects of AFC exposure in children [6]. Because infants and children eat more in proportion to their body weight and their nervous systems are rapidly developing, they are more vulnerable to pre- and postnatal chemical exposure compared with adults [7,8]. Several human studies have reported transient increases in hyperactivity among children after consuming artificial food dyes [9,10]. For example, in 2007, McCann and colleagues conducted a landmark randomized controlled trial in children and observed associations between the intake of several FD&C color additives and hyperactivity-related symptoms [11]. Since then, studies of AFC exposure in the United States have found that children tend to consume products with greater AFC content than adults [12,13,14] and have higher exposure compared to adults on a per-bodyweight basis. To date, no epidemiologic studies have examined prenatal FD&C AFC exposure despite animal studies that suggest behavioral deficits in offspring may result from intake during pregnancy [15].

The total amount of FD&C color additives manufactured for the U.S. market has increased steadily since the mid-1950s [12,16], suggesting the possibility of a higher population intake over time. For example, U.S. food dye production increased from approximately 10 mg/person/day in 1955 to 66 mg/person/day in 2010 [16]. However, processed foods manufactured with U.S.-produced dyes may be exported and foreign products with AFCs may be imported; FD&C color additives are also commonly used in non-food products, such as medications, vitamins, and cosmetics [4], creating uncertainty about domestic per capita use and consumption.

In 2011, the FDA’s Food Advisory Committee recommended additional research to thoroughly examine FD&C batch-certified food dye consumption in the U.S. In response [17], the U.S. FDA conducted a comprehensive AFC exposure study that combined measurements of the seven FD&C food colors in approximately 600 private-label and brand-name foods with short-term food consumption data from the 2007–2010 National Health and Nutrition Examination Survey (NHANES) and longer-term (10–14 days) consumption information for 2007–2010 provided by the NPD Group, Inc. National Eating Trends- Nutrient Intake Database (NPD NET-NID) [14]. Doell et al. estimated dietary exposure to the seven food dyes approved for general use in food in the United States for the U.S. population (aged 2 years and older), children (aged 2–5 years), and teenage boys (aged 13–18 years) based on laboratory measurements of the FD&C color additives in foods [14].

We expanded on the work reported by Doell et al. (2016) to focus on AFC exposure in vulnerable populations, particularly pregnant women, women of childbearing age (18–49 years), and children including infants [14]. This work was part of a larger health risk assessment of food dye exposure by the California Office of Environmental Health Hazard Assessment [9]. Specifically, we used the measurements of the food dye contents of specific foods from Doell et al. 2016 and two-day dietary recall data from the NHANES 2015–2016 survey to estimate intake (mg/kg/day) under two scenarios, typical-exposure, and high-exposure, and calculated both the single-day estimate to approximate acute exposure, and also the two-day average intake [14]. We compared the single-day and two-day average food dye intake estimates to the acceptable daily intake (ADI) values established by the U.S. FDA and the Joint FAO/WHO Expert Committee on Food Additives (JECFA). Finally, because it is well-established that diet patterns and food quality differ by socioeconomic status (SES) in the U.S. [18], we also examined whether women’s and children’s total food dye intake estimates differed by ethnicity, family income, or education.

## 2. Materials and Methods

### 2.1. Food Intake Information

We assessed AFC exposure using food and beverage dietary intake information collected from participants in the 2015–2016 NHANES. The NHANES program assesses the health and nutrition status of children and adults living in the United States by surveying a nationally representative sample of approximately 5000 individuals. Details of the survey methods are described elsewhere [19]. Briefly, participants were asked to recall the specific foods and respective quantities consumed in the 24-h period prior to their in-person interview. A second 24-h recall dietary interview was then scheduled and conducted 3 to 10 days later. Not all participants completed the second interview. An 8-digit U.S. Department of Agriculture (USDA) food code, linked to information about the type of product and brand, was assigned by NHANES to each reported food. We then used the food code to link the consumption data with the AFC concentrations in food reported in the Doell et al. (2016) supplemental data tables [14]. We also extracted information on age, sex, pregnancy status, ethnicity, family income, education, and weight from the survey’s demographics, income, and body measurement datasets [20,21] and merged this information with the dietary recall information [22]. The NHANES survey weights were applied to account for the variable probabilities of selection and non-response of participants to ensure that the results were representative of the U.S. population [19,22]. All data analyses were performed using STATA statistical software Version 15.1 (StataCorp LLC, College Station, TX, USA) [23] and R statistical software version 3.6.3 (R Development Core Team, Vienna, Austria) [24].

### 2.2. Concentrations of FD&C Colors in Food

Concentrations of AFCs in a broad cross-section of foods were measured by Doell and colleagues for the FDA exposure assessment [14,25]. Details of the food selection and laboratory methods are described elsewhere [14,25]. Briefly, Doell et al. (2016) first identified foods and beverages believed to contain at least one AFC based on ingredient lists and a survey of product labels in the Washington D.C. area. Over a two-year period (2012–2014), they surveyed six different stores and over 7300 private-label and brand-name foods [14]. Foods and beverages from the product label survey and databases were then grouped into 52 broad categories (e.g., baby foods, breakfast cereals, cookies, juice drinks, soft drinks, yogurt, etc.), and representative foods from those food categories, based on the results of the product label survey and the information obtained from the available databases and websites, were acquired and analyzed for concentrations (mg/kg) of each of the seven AFCs [25]. For quality control, every 20th sample was analyzed in triplicate. Using the methods adopted by Doell et al. 2016, we assigned the same USDA food codes that most closely matched the NHANES description [14]. If the concentration of an AFC listed as an ingredient in a food was below the limit of detection (LOD) of 1.0 mg/kg, we assumed the AFC was present in the product at the LOD, again following the practices of Doell et al. 2016 [14].

### 2.3. Food Dye Exposure Assessment and Comparison to ADIs

We merged the NHANES food consumption, demographic, and bodyweight data with the AFC concentration data by USDA food code to produce the analytic dataset [20,22]. To compute the AFC intake estimates from a given food, we first converted the self-reported serving size of the food eaten to kilograms and then multiplied this weight by the concentration (mg/kg) of each AFC found in the food. These values were then divided by the individual’s body weight to produce the AFC intake estimates in units of mg/kg/day.

We calculated the single-day (Days 1 and 2) and two-day average daily AFC intake estimates (mg/kg/day) for the following demographic categories: pregnant women 18 years and older; women of childbearing age (18–49 years); and children categorized into the following age groupings: <2 years, 2–<5 years, 5–<9 years, 9–<16 years, and 16–18 years. The single-day AFC intake estimates (Days 1 and 2) were calculated by summing the intake estimates for a given individual and AFC separately for the first and second days of dietary recall. For individuals with two days of dietary recall data, two-day average intake estimates were computed by averaging the single-day estimates. Individuals with two-day average intake estimates consumed an AFC on one or both days of dietary recall (Day 1 and/or Day 2); therefore, the sample sizes for the two-day average intake estimates were sometimes larger than the sample sizes for the single-day estimates. Consistent with Doell et al. (2016), the AFC exposure estimates were produced for “eaters-only” of a given dye, meaning only those individuals consuming at least one food containing the dye were included in the exposure estimate generated for that dye [14].

Two exposure scenarios were examined, as defined in Doell et al. (2016): typical- and high-exposure [14]. The typical-exposure scenario was calculated as follows: (1) for those foods whose AFC content Doell et al. (2016) measured in triplicate, the average of the 3 measurements for each dye was used, and (2) in cases where a single NHANES food code represented multiple foods with distinct dye profiles, the average of the dye concentration values across all foods with that code for each dye were assigned to that food code [14]. The typical-exposure scenario represents the estimated exposure to a given FD&C color for a typical consumer, an individual who may not always eat products with the lowest or highest levels of the FD&C color but some combination of both. The high-exposure scenario estimate was calculated as follows: (1) for those foods whose AFC content was measured in triplicate, the highest of the 3 measurements for each dye was used; and (2) in cases where a single food code represented multiple foods with distinct dye profiles, the maximum concentration of each dye found when looking across all those foods was assigned to that food code. The high-exposure scenario represents the highest possible exposure estimate, where the individual is only consuming products with the highest levels of that AFC.

We calculated the mean, median, and 95th percentile exposure estimates (mg/kg/day) for each AFC (seven in total), exposure scenario (typical and high), demographic category (women of childbearing age, pregnant women, and various age groups of children), and exposure period (Day 1, Day 2, or two-day average). We then compared the mean and 95th percentile single- and two-day intake estimates under each exposure scenario to the established FDA and JECFA ADIs for each AFC (See Supplementary Materials  Table S1) by calculating the ratio of the exposure estimate to the ADI; we describe this ratio as the “hazard ratio” [17,26,27,28,29]. Hazard ratios greater than 1 indicate that the AFC exposure estimate exceeded the established ADI.

### 2.4. Food Category Contributions

Under the typical-exposure scenario, we quantified the contributions of specific food categories to children’s average two-day intake estimates of individual AFCs. Food categories were defined using the short descriptions of USDA food codes found in the NHANES dietary data. For a given AFC and children’s age group, food consumption was grouped by food category and the two-day average exposure to the AFC of interest (typical-exposure scenario) was summed within each food category to produce the total estimated exposure to the AFC attributable to the food category. This quantity was divided by the total exposure to the AFC of interest to generate the percentage of total exposure to the AFC attributable to the food category. Given the large number of food categories, we chose to report a maximum of 10 top contributors for a given AFC and in some cases collapsed similar food categories (e.g., “soft drink, fruit flavored, caffeine containing”, and “soft drink, fruit flavored, caffeine free” were combined as “soft drinks”). For AFCs with <10 contributing food categories, all available food categories were presented.

### 2.5. Ethnicity and Socioeconomic Status Analyses of Total AFC Exposure

We examined the association of several categorical measures of race/ethnicity and SES (i.e., income and education) from the 2015–16 NHANES survey with the two-day average total AFC intake estimates (typical-exposure scenario) in women of childbearing age and children < 18 years old [20,21]. This analysis was limited to AFC “eaters”, i.e., individuals who consumed at least one food containing an AFC on either Day 1 or Day 2 of the dietary recall. The total AFC intake (mg/kg/day) was calculated as the sum of each individual’s seven AFC-specific two-day average intakes (typical-exposure scenario). We used the U.S. Department of Health and Human Services’ (DHHS) federal poverty guidelines (FPG) to define poverty [21,30]. The poverty guidelines are specific to family size, year, and state. We dichotomized the poverty index as ≤130% of the FPG or >130% of the FPG. We defined five race/ethnicity groups from the NHANES categories: Mexican American/other Hispanic; non-Hispanic White; non-Hispanic Black; non-Hispanic Asian, and other race/multiracial. Level of education was constructed as a binary variable: “high school graduate/general education diploma (GED) or less” versus “some college/associate degree or more”. Individuals with missing data for a given variable were excluded from the analysis of that variable.

Because AFC intake estimates were log-normally distributed, we natural log-transformed exposure and then used linear regression to examine the univariate associations between the race/ethnicity, income, and education variables and exposure. Reference groups for the respective analyses were greater than 130% of the FPG; non-Hispanic Whites; and greater than a high school education. Lastly, to facilitate the interpretation, we converted the β coefficients to measurements of the percentage difference in AFC intake associated with a one-unit increase (continuous variables) or a yes/no difference (indicator variables) in the predictor variable using the formula percent difference = 100 × (antilog (β)−1) [31]. We considered *p* < 0.05 for the two-tailed Wald tests of the coefficients to be statistically significant.

## 3. Results

### 3.1. Study Population

The demographic characteristics of the AFC eaters are presented in Table 1. The study sample consisted of 2665 children (18 years), 1224 women of childbearing age, and 51 pregnant women for a total of 3940 individuals. Less than half the children < 2 years were AFC eaters, whereas AFC eaters comprised about 90% of children in other age groups (range: 88.9–95.1%). The prevalence of AFC eaters was greater in women of childbearing age (87.8%) than in pregnant women (74.9%). Generally, non-Hispanic Whites were the most common race/ethnic group among AFC eaters, followed by Mexican Americans/other Hispanics, non-Hispanic Blacks, non-Hispanic other race/multiracial, and non-Hispanic Asians. Among pregnant women, however, non-Hispanic Blacks were slightly more represented (25.7%) than Mexican Americans/other Hispanics (24.0%). Forty-four percent of children aged < 2 were from households with incomes < 130% of federal poverty guidelines. This percentage was lower in the other age groups (range: 30.7–37.2%).

### 3.2. Food Dye Exposure Assessment

Among the seven commonly used FD&C food dyes, the highest estimated exposures for AFC “eaters” were to Red No. 40 followed by Yellow No. 6 (SM Table S6) and Yellow No. 5 (SM Table S5), whereas the estimated Red No. 3 intake exceeded the JECFA ADI in some cases (see below). Table 2 and Table 3 present the single- and two-day average FD&C Red No. 40 and Red No. 3 intake estimates, respectively, for pregnant women, women of childbearing age, and children (0–18 years). Overall, children’s estimated exposure to FD&C Red No. 40 and Red No. 3 as well as the other five FD&C food dyes tended to be higher compared with adult women (Table 2 and Table 3 and SM Tables S2–S6). The range of women’s and children’s (0–18 years) 95th percentile two-day average intake estimates for all seven AFCs ranged from 0.001 to 0.52 mg/kg/day and 0.001 to 0.90 mg/kg/day, respectively (typical-exposure scenario). The highest median Red No. 40 single-day and two-day average intake estimates (mg/kg/day) were observed for children 5–<9 years old (0.21 and 0.17 mg/kg/day, respectively) (typical-exposure scenario). For the high-exposure scenario, the highest median FD&C Red No. 40 single-day and two-day average estimated intakes were also found in children 5–<9 years old (0.39 mg/kg/day and 0.32 mg/kg/day, respectively). The highest 95th percentile single-day dose estimates based on the average- and high-exposure scenarios, however, were found for FD&C Red No. 3 in children < 2 years (4.83 mg/kg/day and 7.90 mg/kg/day, respectively) (Table 3). The lowest exposures were found for Green No. 3 (SM Table S4).

### 3.3. Comparison of FD&C Food Dye Intake Estimates with ADIs

Table 3 presents the ratios of mean and 95th percentile single- and two-day average Red No. 3 exposure (mg/kg/day) to the FDA and JECFA ADIs. Exceedances of the JECFA and FDA ADIs were observed for Red No. 3 under both the typical- and high- exposure scenarios but not for the other AFCs. The ADIs for Red No. 3 established by the JECFA (0.1 mg/kg/day) and the FDA (2.5 mg/kg/day) differ by more than an order of magnitude due to the different studies used by the agencies to establish their ADIs. The FDA ADI for Red No. 3 was based on two-year toxicological studies in rats and dogs conducted by the FDA from 1952 to 1954 and the JECFA ADI was based on a 14-day study in 30 men published in 1987 that reported an increase in thyroid-stimulating hormone (TSH) responsiveness [9,17,32].

Under the typical-exposure scenario, mean single-day exposure estimates (mg/kg/day) for children < 2 years, children 2–<5 years, and children 5–<9 years exceeded the JECFA ADI (ADI = 0.1 mg/kg/day), with hazard ratios ranging from 1.1 to 5.4 (Table 3). Exceedances were also observed for the 95th percentile single-day estimates for pregnant women, women of childbearing age, children < 2 years, children 2–<5 years, children 5–<9 years, children 9–<16 years, and youth 16 to 18 years, with hazard ratios ranging from 1.0 to 48.3. Only children <2 years were in exceedance of the JECFA ADI for Red No. 3 (ADI = 0.1 mg/kg/day) based on the mean two-day exposure estimates, with a hazard ratio of 1.73. Children 5–<16 years were all in exceedance of the ADI based on the 95th percentile two-day average exposure estimates. The high-exposure scenario estimates followed similar trends with the exception of the mean two-day average estimates for children 2–<5 years and 9–<16 years, which also exceeded the ADI. Children < 2 years were the only group to exceed the FDA ADI (ADI = 2.5 mg/kg/day), with typical- and high- exposure scenario single-day 95th percentile estimates exceeding the ADI by 1.93- and 3.16-fold, respectively.

Women’s and children’s food dye intake estimates for FD&C Blue No. 1, Blue No. 2, Green No. 3, Red No 40, Yellow No. 5, and Yellow No. 6 compared to their FDA and JECFA ADIs are presented in the Supplementary Materials (Tables S2–S6). None exceeded their respective FDA or JECFA ADIs.

### 3.4. Major Food Category Contributors to AFC Exposure

**Figure 1:**
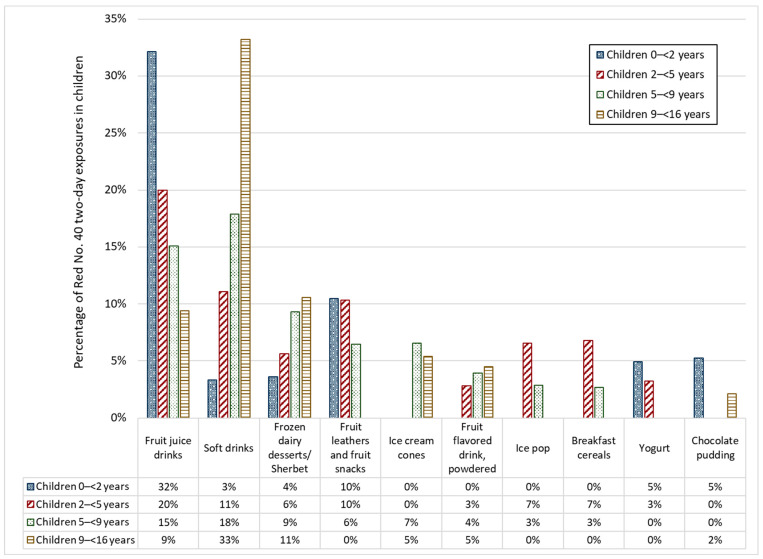
Top foods contributing to FD&C Red No. 40 two-day average exposure estimates in children aged 0–<16 years (typical-exposure scenario) who consumed at least one food containing Red No. 40.

**Figure 2:**
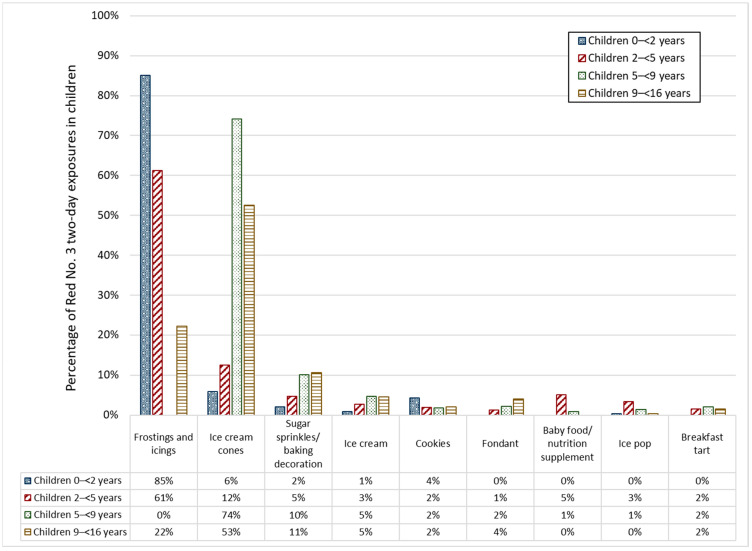
Top foods contributing to FD&C Red No. 3 two-day average exposure estimates in children aged 0–<16 years (typical-exposure scenario) who consumed at least one food containing Red No. 3.

Figure 1 and Figure 2 present the top food categories that contributed to children’s estimated two-day average FD&C Red No. 40 and Red No. 3 intakes (typical-exposure scenario), respectively. Overall, fruit juice drinks and soft drinks were important sources of exposure to FD&C Red No. 40 for all children; however, this contribution was exceeded by soft drinks in children 9–<16 years old (Figure 1). The primary contributors of FD&C Red No. 3 were frosting and icings and ice cream cones for children 0–<5 years and children 5–<16 years, respectively (Figure 2). In children 5–<9 years, 74% of Red No. 3 exposure was attributable to ice cream cones. Children 9–<16 years and 2–<5 years received 52% and 12%, respectively, of their Red. No. 3 exposure from ice cream cones. Children < 2 received the least exposure from ice cream cones, with 5% of their Red No. 3 exposure attributable to this food category. Frostings and icings were the sources of 85%, 61%, and 22% of Red No. 3 exposure in children <2 years, 2–<5 years, and 9–<16 years, respectively. This food category includes decorating gels, which contain high concentrations of FD&C food color additives. Similar top food category trends were observed for the other dyes (see Supplementary Materials  Figures S1–S5).

### 3.5. Association of Artificial Food Color Intake with Socioeconomic Variables

**Figure 3:**
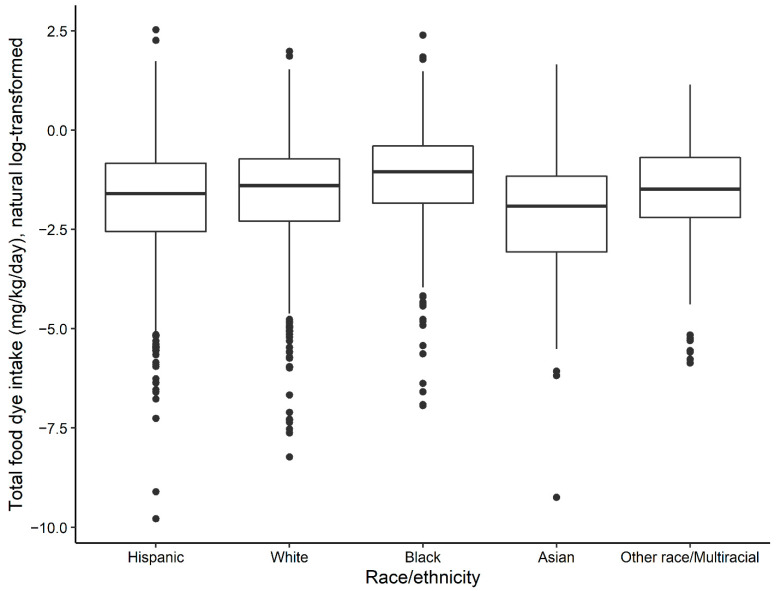
Total two-day average food dye intake estimates (natural log-transformed mg/kg/day) for children (0–18 years) by ethnicity (n = 2203).

The results from linear regression models examining the associations of total estimated AFC intake (mg/kg/day) with ethnicity and SES are presented in Table 4. All estimates are based on AFC “eaters” only. We found significant, positive associations between measures of poverty, education, and ethnicity with the total estimated two-day average food dye intake (typical-exposure scenario) for children 0–18 years and women of childbearing age who consumed at least one food containing an AFC. Among women, belonging to a household with a monthly income of ≤130% of the FPG was associated with an estimated increase of 42% (95% CI: 6.0, 89.9) in total AFC intake compared with having a household income greater than 130% of FPG. However, the percentage of eaters in the high-income group (89.1%) was slightly greater compared to the low-income group (85.1%). Black children consumed 71% (95% CI: 31.1, 123.6) more AFCs compared with White children (Figure 3). Disparities in the AFC intake by ethnicity in women of childbearing age were similar to those seen in children but were of a lesser magnitude. Cumulative distribution graphs representing the total estimated two-day average food dye intake (typical-exposure scenario) for women and children by ethnicity are presented in the Supplementary Materials ( Figures S6 and S7). In addition, women AFC “eaters” with a high school education or less had a 72% (95% CI: 14.0, 159.2) higher AFC intake compared with those with >high school education (Table 4).

The estimated total food dye intake was significantly higher among non-Hispanic Black children compared to non-Hispanic White children who consumed at least one food containing an AFC (linear regression *p*-value = 0.001).

Children: Mexican American/Hispanic (n = 722); non-Hispanic White (n = 666); non-Hispanic Black (n = 521); Non-Hispanic Asian (n = 152) and other race/multiracial (n = 142).

Box-and-whisker plot notation: the middle bar represents the median; the bottom and top of the box represent the 25th and 75th percentiles; the bottom and top of the whiskers represent the 25th percentile minus 1.5 times the interquartile range (75th percentile minus 25th percentile), and the 75th percentile plus 1.5 times the interquartile range, respectively; and the dots represent potential outliers.

## 4. Discussion

In this study, we assessed the dietary intake of artificial food dyes in women and children living in the United States. The highest exposure estimates for both groups were to FD&C Red No. 40, followed by Yellow No. 6 and Yellow No. 5. Children’s and pregnant women’s single-day and/or two-day average FD&C Red No. 3 intake estimates exceeded the JECFA ADI (0.1 mg/kg/day) for both “typical” and “high” exposure scenarios in several instances. The single-day intake estimates for children < 2 years old also exceeded the U.S. FDA ADI (2.5 mg/kg/day) in one instance. Fruit juice drinks, soft drinks, frostings and icings, and ice cream cones were the major food categories contributing to children’s (<16 years old) exposure to multiple FD&C color additives.

To our knowledge, this study is the first to estimate AFC intake from food and beverages in young children (<2 years) in the U.S. (the youngest age group reported in previous U.S. studies was for children 2–5 years [14,33]). AFC exposure assessments conducted in other countries, including India [34] and Korea [35], have reported intakes for infants that in some cases exceeded the JECFA ADI. We found infants to have AFC intakes comparable to children of other ages for all dyes, which in some instances also exceeded the JECFA ADI for Red No. 3 and, under one scenario, the FDA Red No. 3 ADI. These estimates, however, were generated among individuals who consumed at least one food containing an AFC. Among children < 2 years old, we found that the “eaters” comprised just 44.5% of the total population surveyed in this age group compared to 85% of children in older age categories. Thus, the overall AFC exposure burden is less in children < 2 years old compared to older children. In general, we found that food dye exposures tended to be higher in children compared to women of childbearing age. This finding is consistent with prior work suggesting that children consume AFC-containing products more often than adults [12,13,14].

To estimate food dye intake, we applied methods developed by the U.S. FDA [14] but extended them to include pregnant women and young children. In both studies, Red No. 40, Yellow No. 5, and Yellow No. 6 showed the highest intakes. We used more recent NHANES food consumption data (2015–2016 versus 2007–2010) but overall, the estimated AFC intake estimates were not substantially different from Doell et al. For the most comparable age category in both studies (children 2 to 5 years), the mean two-day average Red No. 40 intake estimates were similar for both typical- and high- exposure scenarios [14], suggesting that food consumption patterns had not markedly changed between the NHANES surveys used by Doell and this study. Bastaki et al. (2017) conducted a similar study using NHANES dietary data but used different information to assess the AFC presence in foods (e.g., use of industry surveys rather than AFC measurements), resulting in lower estimates of AFC intake. Our reliance on laboratory measurements published by the FDA and recent national food consumption information provides an up-to-date assessment of current U.S. population exposures. We did not use the approach taken by Bastaki et al. (2017) for our assessment because the industry surveys used may not have been exclusive to the U.S. and the use of a proprietary database of finished product labels possibly underestimated the frequency with which food labels listed FD&C color additives as ingredients.

We found that fruit juice drinks were major contributors to Red No. 40 intake in children of all ages; however, soft drinks were the dominant contributor among children 9–<16 years old. This finding is consistent with Doell et al. The major contributors to Red No. 3 intake in our study overlap somewhat with what was reported by Doell and colleagues [14]. Specifically, both studies found that ice cream cones were major contributors to Red No. 3 intake in children 2–<5 years. In contrast, a substantial fraction of the intake in children of this age group was attributable to frostings and icings in our study, whereas Doell et al. found a somewhat smaller share for these sources. Future studies should assess the trends in AFC consumption.

To our knowledge, this is the first report examining the associations between the SES indicators, ethnicity, and AFC intake in the United States. Overall, our analysis suggests some trends with higher exposure in lower-income families with less education, and higher intake among non-Hispanic Black participants compared with other ethnic groups (Hispanic, non-Hispanic White, and Asian or other categories). It is possible that the availability of food products in different neighborhoods in the U.S. may impact exposure. For example, markets in regions where people have limited access to healthful and affordable food, such as food deserts in lower-income communities, might not carry the same range of products available in more affluent communities, thus limiting choices [36,37]. Also, some U.S. supermarket chains, often the more expensive ones, have explicit policies prohibiting the sale of foods containing artificial food colorings. As a result, consumers without access to these stores may have higher exposure because their neighborhood markets are more likely to sell foods containing AFCs even if they are purchasing the same general food categories as consumers in other neighborhoods. Thus, the differences in exposure associated with socioeconomic variables may, in part, be due to food systems that unevenly distribute AFC-containing products to communities. Given that foods containing AFCs are generally less healthy (i.e., highly processed, high sugar content, etc.), our results support the hypothesis that AFC intake may be an indicator of poorer diet and related health disparities [38].

The current study has several strengths. We utilized a large, representative sample from NHANES to obtain single- and two-day dietary recall information. We used U.S. FDA-supervised laboratory measurements of over 600 foods as the basis of our assessment [14,25]. Examining food dye consumption over two days represents a short-term intake, which may have clinical relevance for neurobehavioral outcomes resulting from children’s exposure to AFCs [9,10,11,39,40,41]. Lastly, we calculated maternal and early postnatal exposure estimates and structured the child age categories to allow for intake estimations through the developmentally distinct phases of childhood.

This study also has several limitations. We were not able to control for potential confounding variables in our analysis of total food dye intake and measures of ethnicity and SES. Temporal differences between the NHANES food consumption data collection and the food dye laboratory measurements may have also introduced uncertainties. For example, it is possible that AFC use in foods has declined or increased since the FDA completed its laboratory measurements; overall, U.S. food dye production per capita has been increasing steadily [12,15]. Although the FDA data set of AFC concentrations in food is the most comprehensive in the world, some AFC-containing foods may have been missed, resulting in underestimates of exposure. Additionally, measurements by other laboratories to independently confirm the FDA-reported values would improve the reliability of the exposure estimates. Of note, the California Office of Environmental Health Hazard Assessment (OEHHA) commissioned additional limited measurements of AFCs in food by a separate laboratory that reported concentrations within the range of the FDA results [9]. We found that frostings and icings contributed to children’s AFC exposure. This food category includes decorating gels that may contain high concentrations of FD&C food color additives. Future studies should further evaluate the contribution of these products to the total AFC exposure. Also, our exposure assessment did not distinguish between the intake of AFC straights versus lakes. Limited information is available on the proportion of AFCs that is absorbed by the gut and there may be differences between straights and lakes. For epidemiological and toxicological studies and conducting risk assessments, distinguishing exposure to different AFC forms may improve study designs and better inform regulations governing exposure. Although our utilization of the NHANES survey weights should have largely accounted for non-responses on the second day of dietary recall data, loss to follow-up may have increased uncertainty in our Day 2 AFC intake estimates. Finally, the two-day NHANES food consumption data did not allow us to assess chronic or sub-chronic exposures.

Overall, most of our AFC exposure estimates were below the U.S. FDA and JECFA ADIs, although we found that some U.S. children and pregnant women may consume FD&C Red No. 3, at least for short periods, at levels that exceed intake guidelines. However, these ADIs are mostly based on reports of general toxicity in older studies that lacked the power to detect neurobehavioral outcomes, especially in children. Moreover, the ADIs do not reflect more recent animal and human studies that suggest adverse behavioral outcomes associated with AFC exposure [9].

## 5. Conclusions

AFC intake from food is common across different age groups in the U.S., with patterns of higher exposure in children compared with adults. Children are also exposed to AFCs through over-the-counter medications and vitamins [42,43]. Future studies should quantify total AFC exposure for pregnant women and children over time based on comprehensive laboratory measurements of food and pharmaceuticals to better characterize the exposure and potential health risks. Given that AFCs are found in processed foods high in refined carbohydrates and of low nutritional value, trends in higher exposure in lower-income families with less education, and higher intake among non-Hispanic Black participants compared with other ethnic groups, underscore the need for more research on the sociodemographic determinants of AFC exposure, diet quality, and interventions to improve access to healthier food. Additionally, to improve the estimates of internal doses, research is needed to understand the absorption of the dyes by the gut, including the differences in absorbed doses resulting from the intake of straight and lake AFCs.

## Figures and Tables

**Table 1:** Demographic characteristics of AFC eaters (children and women of childbearing age) who consumed at least one AFC on Day 1 and/or Day 2 (n = 2665 children and 1275 adult eaters, U.S.).

	Children					Women	
	Age < 2	Age 2–<5	Age 5–<9	Age 9–<16	Age 16–18	Age 18–49	Pregnant
N (% eaters) ^a^	217 (44.5)	443 (90.0)	618 (95.1)	1005 (91.8)	382 (88.9)	1224 (87.8)	51 (74.9)
Age (years; mean (SD))	0.8 (0.4)	3.0 (0.8)	6.6 (1.1)	12.1 (2.0)	16.9 (0.8)	34.2 (9.1)	28.9 (5.7)
Gender (N (%))							
Female	107 (44.5)	219 (58.7)	301 (47.0)	486 (47.1)	207 (58.8)	-	-
Male	110 (55.5)	224 (41.3)	317 (53.0)	519 (52.9)	175 (41.2)	-	-
Race (N (%))							
Mexican American/Other Hispanic	70 (29.2)	132 (23.5)	214 (27.9)	333 (24.7)	125 (24.9)	393 (20.1)	18 (24.0)
Non-Hispanic White	77 (51.6)	153 (53.3)	181 (47.8)	301 (48.6)	104 (51.3)	347 (55.2)	12 (37.0)
Non-Hispanic Black	44 (12.8)	106 (14.1)	147 (15.4)	231 (15.2)	97 (16.6)	306 (14.1)	13 (25.7)
Non-Hispanic Asian	7 (1.0)	20 (3.3)	28 (2.8)	82 (4.8)	36 (4.0)	126 (6.3)	4 (3.8)
Non-Hispanic Other Race/Multiracial	19 (5.4)	32 (5.9)	48 (6.2)	58 (6.8)	20 (3.2)	52 (4.3)	4 (9.4)
Income (%)							
≤130% FPG	93 (44.5)	198 (33.6)	270 (32.1)	427 (37.2)	161 (34.6)	460 (30.7)	17 (34.2)
>130% FPG	115 (53.8)	220 (63.7)	325 (66.2)	530 (61.2	192 (64.7)	695 (66.9)	29 (64.6)
Don’t know	4 (1.7)	5 (2.2)	10 (1.6)	8 (1.7)	7 (0.6)	24 (1.6)	1 (1.2)
Declined to answer	0 (0)	1 (0.5)	0 (0)	1 (0)	2 (0.1)	5 (0.8)	0 (0)
Body weight (kg) (mean (SD))	10.8 (1.5)	16.2 (3.4)	26.1 (7.3)	52.2 (19.0)	72.4 (21.3)	77.4 (20.7)	79.0 (17.7)

Abbreviation: FPG (federal poverty guidelines), AFC eaters: individuals reporting dietary data for one or two days who ate at least one food containing any of the seven FD&C artificial food dyes. ^a^ “% eaters” represents the percentage of a subpopulation that consumed at least one product containing an AFC over the dietary recall period; it was calculated as the number of individuals in the subpopulation who reported consuming at least one food containing an AFC divided by the total number of individuals in the subpopulation and multiplied by 100%.

**Table 2:** Single-day and two-day average FD&C Red No. 40 exposure estimates (mg/kg/day) and hazard ratios among pregnant women, women of childbearing age, and children of various ages who consumed at least one food containing Red No. 40.

Red No. 40			Typical-Exposure Scenario							High-Exposure Scenario						
						FDA Ratio ^d^		JECFA Ratio ^d^					FDA Ratio ^d^		JECFA Ratio ^d^	
	Total n ^b^	n ^c^	Mean	Median	95th%	Mean	95th%	Mean	95th%	Mean	Median	95th%	Mean	95th%	Mean	95th%
Pregnant women																
Day 1	48	44	0.14	0.04	0.53	0.02	0.08	0.02	0.08	0.26	0.07	1.38	0.04	0.20	0.04	0.20
Day 2	31	27	0.08	0.01	0.31	0.01	0.04	0.01	0.04	0.24	0.03	1.72	0.03	0.25	0.03	0.25
2-Day average ^a^	42	39	0.09	0.03	0.52	0.01	0.07	0.01	0.07	0.21	0.06	0.69	0.03	0.10	0.03	0.10
Women 18–49 years																
Day 1	1048	982	0.11	0.05	0.37	0.02	0.05	0.02	0.05	0.23	0.08	0.91	0.03	0.13	0.03	0.13
Day 2	792	722	0.10	0.05	0.35	0.01	0.05	0.01	0.05	0.26	0.08	1.20	0.04	0.17	0.04	0.17
2-Day average ^a^	1040	979	0.08	0.04	0.28	0.01	0.04	0.01	0.04	0.19	0.06	0.70	0.03	0.10	0.03	0.10
Children (<2 years)																
Day 1	177	166	0.29	0.13	1.01	0.04	0.14	0.04	0.14	0.57	0.22	2.65	0.08	0.38	0.08	0.38
Day 2	131	121	0.25	0.12	1.00	0.04	0.14	0.04	0.14	0.51	0.17	2.11	0.07	0.30	0.07	0.30
2-Day average ^a^	186	175	0.20	0.08	0.90	0.03	0.13	0.03	0.13	0.40	0.11	1.69	0.06	0.24	0.06	0.24
Children (2–<5 years)																
Day 1	388	366	0.30	0.16	0.91	0.04	0.13	0.04	0.13	0.66	0.23	3.28	0.09	0.47	0.09	0.47
Day 2	300	265	0.30	0.18	0.92	0.04	0.13	0.04	0.13	0.73	0.32	3.02	0.10	0.43	0.10	0.43
2-Day average ^a^	363	352	0.23	0.13	0.75	0.03	0.11	0.03	0.11	0.52	0.25	2.04	0.07	0.29	0.07	0.29
Children (5–<9 years)																
Day 1	569	550	0.30	0.21	0.91	0.04	0.13	0.04	0.13	0.71	0.39	2.51	0.10	0.36	0.10	0.36
Day 2	397	378	0.26	0.17	0.79	0.04	0.11	0.04	0.11	0.73	0.27	2.97	0.10	0.42	0.10	0.42
2-Day average ^a^	501	491	0.23	0.17	0.73	0.03	0.10	0.03	0.10	0.60	0.32	2.13	0.09	0.30	0.09	0.30
Children (9–<16 years)																
Day 1	908	860	0.20	0.14	0.63	0.03	0.09	0.03	0.09	0.52	0.25	2.05	0.07	0.29	0.07	0.29
Day 2	660	622	0.20	0.13	0.68	0.03	0.10	0.03	0.10	0.56	0.23	2.72	0.08	0.39	0.08	0.39
2-Day average ^a^	843	822	0.16	0.11	0.51	0.02	0.07	0.02	0.07	0.44	0.23	1.63	0.06	0.23	0.06	0.23
Youth (16–18 years)																
Day 1	342	315	0.13	0.08	0.43	0.02	0.06	0.02	0.06	0.30	0.12	1.18	0.04	0.17	0.04	0.17
Day 2	222	201	0.11	0.05	0.35	0.02	0.05	0.02	0.05	0.28	0.07	1.08	0.04	0.15	0.04	0.15
2-Day average ^a^	310	301	0.09	0.05	0.29	0.01	0.04	0.01	0.04	0.21	0.08	0.82	0.03	0.12	0.03	0.12

Abbreviations: 95th%: 95th percentile; FDA: Food and Drug Administration; JECFA: Joint FAO/WHO Expert Committee on Food Additives. Note: If FD&C Red No. 40 was listed on the label for a food but the results for that color additive were below the LOD, we assumed that the AFC was present in the product at the LOD (i.e., 1.0 mg/kg). ^a^ The 2-day average estimates include individuals who completed both the Day 1 and Day 2 NHANES food consumption questionnaires and consumed a food containing Red No. 40 on one or both of those days. ^b^ Total n = number of AFC eaters, i.e., individuals who ate at least one food containing any of the seven FD&C artificial food dyes. ^c^ n = number of individuals who consumed at least one food containing FD&C Red 40; means, medians, and 95th percentiles are calculated based on these individuals. ^d^ Ratio of mean and 95th percentile single-day and two-day average FD&C Red No. 40 exposure (mg/kg/day) to FDA or JECFA ADIs. The FDA and JECFA ADI for FD&C Red No. 40 is 7 mg/kg/day.

**Table 3:** Single-day and two-day average FD&C Red No. 3 exposure estimates (mg/kg/day) and hazard ratios under typical- and high-exposure scenarios, among pregnant women, women of childbearing age, and children of various ages who consumed at least one food containing Red No. 3.

Red No. 3			Typical-Exposure Scenario							High-Exposure Scenario						
						FDA Ratio ^d^		JECFA Ratio ^d^					FDA Ratio ^d^		JECFA Ratio ^d^	
	Total n ^b^	n ^c^	Mean	Median	95th%	Mean	95th%	Mean	95th%	Mean	Median	95th%	Mean	95th%	Mean	95th%
Pregnant women																
Day 1	48	20	0.03	0.01	0.23	0.01	0.09	0.29	2.28	0.06	0.02	0.67	0.02	0.27	0.60	6.66
Day 2	31	18	0.02	0.02	0.04	0.008	0.02	0.20	0.41	0.02	0.02	0.05	0.01	0.02	0.23	0.54
2-Day average ^a^	42	25	0.02	0.02	0.11	0.008	0.05	0.20	1.14	0.04	0.02	0.33	0.01	0.13	0.35	3.33
Women 18–49 years																
Day 1	1048	520	0.03	0.01	0.08	0.01	0.03	0.27	0.78	0.04	0.01	0.08	0.02	0.03	0.38	0.81
Day 2	792	396	0.03	0.01	0.10	0.01	0.04	0.29	1.02	0.04	0.02	0.10	0.02	0.04	0.38	1.02
2-Day average ^a^	1040	592	0.02	0.007	0.07	0.01	0.03	0.18	0.72	0.02	0.009	0.08	0.01	0.03	0.24	0.80
Children (<2 years)																
Day 1	177	72	0.03	0.02	0.09	0.01	0.04	0.28	0.90	0.03	0.02	0.11	0.01	0.04	0.32	1.11
Day 2	131	53	0.54	0.01	4.83	0.21	1.93	5.35	48.3	1.50	0.01	7.90	0.60	3.16	15.0	79.0
2-Day average ^a^	186	84	0.17	0.008	0.07	0.07	0.03	1.73	0.68	0.47	0.008	0.07	0.19	0.03	4.72	0.68
Children (2–< 5 years)																
Day 1	388	200	0.19	0.01	0.19	0.08	0.07	1.89	1.85	0.49	0.01	0.19	0.19	0.08	4.85	1.90
Day 2	300	126	0.06	0.02	0.16	0.02	0.06	0.56	1.56	0.08	0.02	0.17	0.03	0.07	0.84	1.68
2-Day average ^a^	363	214	0.07	0.008	0.09	0.03	0.04	0.70	0.90	0.17	0.009	0.09	0.07	0.04	1.66	0.90
Children (5–< 9 years)																
Day 1	569	320	0.06	0.009	0.11	0.03	0.04	0.64	1.12	0.11	0.01	0.14	0.04	0.06	1.05	1.38
Day 2	397	209	0.11	0.01	0.20	0.04	0.08	1.09	1.98	0.17	0.01	0.21	0.07	0.09	1.72	2.14
2-Day average ^a^	501	349	0.06	0.007	0.12	0.02	0.05	0.62	1.22	0.10	0.009	0.23	0.04	0.09	0.98	2.28
Children (9–< 16 years)																
Day 1	908	456	0.09	0.008	0.16	0.03	0.06	0.87	1.61	0.20	0.01	0.32	0.08	0.13	1.96	3.19
Day 2	660	303	0.09	0.01	0.14	0.03	0.06	0.87	1.38	0.15	0.01	0.14	0.06	0.06	1.52	1.44
2-Day average ^a^	843	536	0.06	0.007	0.16	0.02	0.06	0.55	1.60	0.11	0.009	0.42	0.04	0.17	1.05	4.21
Youth (16–18 years)																
Day 1	342	130	0.05	0.006	0.21	0.02	0.09	0.49	2.14	0.07	0.007	0.21	0.03	0.09	0.69	2.14
Day 2	222	99	0.02	0.007	0.06	0.007	0.02	0.17	0.57	0.02	0.01	0.08	0.01	0.03	0.21	0.80
2-Day average ^a^	310	162	0.02	0.004	0.05	0.007	0.02	0.18	0.54	0.02	0.006	0.06	0.01	0.02	0.24	0.62

Abbreviations: 95th%: 95th percentile; FDA: Food and Drug Administration; JECFA: Joint FAO/WHO Expert Committee on Food Additives; ADI: acceptable daily intake Note: If FD&C Red No. 3 was listed on the label for a food but the results for that color additive were below the LOD, we assumed that the AFC was present in the product at the LOD (i.e., 1.0 mg/kg). ^a^ The 2-day average estimates include individuals who completed both the Day 1 and Day 2 NHANES food consumption questionnaires and consumed a food containing Red No. 3 on one or both of those days. ^b^ Total n = number of AFC eaters, i.e., individuals who ate at least one food containing any of the seven FD&C artificial food dyes. ^c^ n = number of individuals who consumed at least one food containing FD&C Red 3; means, medians, and 95th percentiles are calculated based on these individuals. ^d^ Ratio of mean and 95th percentile single-day and two-day average FD&C Red No. 3 exposure (mg/kg/day) to FDA or JECFA ADIs. For FD&C Red No. 3, the FDA ADI is 2.5 mg/kg/day and the JECFA ADI is 0.1 mg/kg/day. Ratios > 1 indicate estimated exposures that exceed the ADI.

**Table 4:** Associations between race/ethnicity and measures of socioeconomic status and two-day average total estimated artificial food coloring intake (mg/kg/day) in children and women of childbearing age who consumed at least one food containing an AFC.

	Children (0–18 Years) ^a^			Women of Childbearing Age (18–49 Years) ^a^		
	N (% Eaters) ^b,c^	Geometric Mean Intake (95% CI)	Percent Difference in Intake (95% CI)	N (% Eaters) ^b,c^	Geometric Mean Intake (95% CI)	Percent Difference in Intake (95% CI)
Income						
>130% of FPG ^d,e^	1151 (88)	0.20 (0.18, 0.22)	Ref.	603 (89.1)	0.07 (0.05, 0.08)	Ref.
≤130% of FPG ^d,e^	939 (86.3)	0.24 (0.20, 0.28)	18.8 (−2.9, 45.4)	383 (85.1)	0.09 (0.07, 0.12)	41.8 (6.0, 89.8)
Race/ethnicity						
White, Non-Hispanic	666 (88.8)	0.19 (0.17, 0.22)	Ref.	305 (88.4)	0.07 (0.05, 0.09)	Ref.
Black, Non-Hispanic	521 (90.2)	0.33 (0.28, 0.39)	71.2 (31.1, 123.6)	251 (91.0)	0.11 (0.09, 0.14)	60.8 (16.6, 121.9)
Mexican American/Other Hispanic	722 (85.1)	0.20 (0.16, 0.24)	3.0 (−17.6, 28.8)	330 (88.7)	0.07 (0.05, 0.08)	−3.3 (−32.0, 37.4)
Asian, Non-Hispanic	152 (79.6)	0.13 (0.09, 0.18)	−34.5 (−57.9, 2.1)	109 (76.7)	0.08 (0.06, 0.10)	12.1 (−21.7, 60.4)
Other race/Multiracial	142 (80.4)	0.25 (0.17, 0.36)	31.7 (−12.0, 96.9)	45 (83.3)	0.04 (0.02,0.07)	−43.0 (−68.5, 3.2)
Education						
More than high school/GED				620 (88.2)	0.06 (0.05, 0.08)	Ref.
High school/GED or less				260 (86.3)	0.10 (0.08, 0.13)	71.9 (14.0, 159.2)

Abbreviations: AFC: Artificial food color; FPG: (federal poverty guidelines); GED (General Education Diploma). ^a^ NHANES survey weights were applied to account for variable probabilities of selection and non-response of participants to ensure the results were representative of the U.S. population. ^b^ “N” represents the number of children or women in each demographic category that consumed at least one food product containing a food dye on Day 1 or Day 2 of the NHANES survey. ^c^ “% eaters” represents the percentage of a subpopulation that consumed at least one product containing an AFC over the dietary recall period; it was calculated as the number of individuals in the subpopulation in the U.S. who reported consuming at least one food containing an AFC divided by the total number of individuals in the subpopulation in the U.S. and multiplied by 100%. ^d^ Based on the 2015 and 2016 U.S. Department of Health and Human Services Federal Poverty Guidelines. ^e^ Participants with missing “income” information were excluded from the regression analysis of that variable.

## Data Availability

This study used publicly available data from the CDC NHANES survey and the U.S. Food and Drug Administration.

## Supplementary Materials

The following supporting information can be downloaded at: https://www.mdpi.com/article/10.3390/ijerph19159661/s1, Table S1: Food dye ADIs established by the U.S FDA and JECFA; Table S2: Estimated single-day and two-day average FD&C Blue No. 1 exposure (mg/kg/day) and hazard ratios under typical- and high-exposure scenarios among pregnant women, women of childbearing age, and children of various ages who consumed at least one food containing Blue No. 1; Table S3: Estimated single-day and two-day average FD&C Blue No. 2 exposure (mg/kg/day) and hazard ratios under typical- and high-exposure scenarios among pregnant women, women of childbearing age, and children of various ages who consumed at least one food containing Blue No. 2; Table S4: Estimated single-day and two-day average FD&C Green No. 3 exposure (mg/kg/day) and hazard ratios under typical- and high-exposure scenarios among pregnant women, women of childbearing age, and children of various ages who consumed at least one food containing Green No. 3; Table S5: Estimated single-day and two-day average FD&C Yellow No. 5 exposure (mg/kg/day) and hazard ratios under typical- and high-exposure scenarios among pregnant women, women of childbearing age, and children of various ages who consumed at least one food containing Yellow No. 5; Table S6: Estimated single-day and two-day average FD&C Yellow No. 6 exposure (mg/kg/day) and hazard ratios under typical- and high-exposure scenarios among pregnant women, women of childbearing age, and children of various ages who consumed at least one food containing Yellow No. 6; Figure S1: Top foods contributing to FD&C Blue No. 1 exposure estimates in children ages 0–< 16 years (Typical-exposure scenario); Figure S2: Top foods contributing to FD&C Blue No. 2 exposure estimates in children ages 0–< 16 years (Typical-exposure scenario); Figure S3: Top foods contributing to FD&C Green No. 3 exposure estimates in children ages 0–< 16 years (Typical-exposure scenario); Figure S4: Top foods contributing to FD&C Yellow No. 5 exposure estimates in children ages 0–< 16 years (Typical-exposure scenario); Figure S5: Top foods contributing to FD&C Yellow No. 6 exposure estimates in children ages 0–< 16 years (Typical-exposure scenario); Figure S6: Children’s (0–18 years) estimated total food dye intake by ethnicity (mg/kg/day); Figure S7: Women’s (18–49 years) estimated total food dye intake by ethnicity (mg/kg/day).
