# Supplementary material for: Dietary Exposure to United States Food and Drug Administration-Approved Synthetic Food Colors in Children, Pregnant Women, and Women of Childbearing Age Living in the United States
Source: Int J Environ Res Public Health. 2022 Aug 5;19(15):9661. doi: 10.3390/ijerph19159661 (PMC9368057; doi:10.3390/ijerph19159661)
Supplement: Supplementary file 1 [file ijerph-19-09661-s001.zip › ijerph-1806988-Supplementary.pdf]

Supplementary Materials

Dietary exposures to United States Food and Drug Administration approved synthetic food colors in children, pregnant women, and women of childbearing age living in the United States

Asa Bradman[1,2*], Rosemary Castorina[1], Ruwan Thilakaratne[1], Mayela Gillan[1], Teja Pattabhiraman[1], Anuroop Nirula[1], Melanie Marty[3], Mark Miller[3]

Authors' affiliation
[1]Center for Environmental Research and Community Health (CERCH), School of Public Health, University of California at Berkeley, 1995 University Avenue, Suite 265, Berkeley, CA 94704
[2]Department of Public Health, School of Social Sciences, Humanities, and Art, University of California, Merced, 5200 N. Lake Road, Merced, CA 95343
[3]California Office of Environmental Health Hazard Assessment, Oakland, CA

---

20 pages, 6 tables, 7 figures

***Corresponding author:**

Asa Bradman, PhD

Department of Public Health

School of Social Sciences, Humanities, and Arts

5200 North Lake Rd.

Merced, CA 95343

abradman@ucmerced.edu

Tables

Table S1. Food dye ADI's established by the U.S FDA and JECFA ………………......……... 3

Table S2. Estimated single-day and two-day average FD&C Blue No. 1 exposure (mg/kg/day) and hazard ratios under typical- and high-exposure scenarios, among pregnant women, women of childbearing age, and children of various ages who consumed at least one food containing Blue No. 1 ………………………………………………………………………………......…. 4

Table S3. Estimated single-day and two-day average FD&C Blue No. 2 exposure (mg/kg/day) and hazard ratios under typical- and high-exposure scenarios, among pregnant women, women of childbearing age, and children of various ages who consumed at least one food containing Blue No. 2 ………………………………………………………………………………....…. 6

Table S4. Estimated single-day and two-day average FD&C Green No. 3 exposure (mg/kg/day) and hazard ratios under typical- and high-exposure scenarios, among pregnant women, women of childbearing age, and children of various ages who consumed at least one food containing Green No. 3 ………………………………………………………………………………......….. 8

Table S5. Estimated single-day and two-day average FD&C Yellow No. 5 exposure (mg/kg/day) and hazard ratios under typical- and high-exposure scenarios, among pregnant women, women of childbearing age, and children of various ages who consumed at least one food containing Yellow No. 5 …………………………………………………………..…………………. 10

Table S6. Estimated single-day and two-day average FD&C Yellow No. 6 exposure (mg/kg/day) and hazard ratios under typical- and high-exposure scenarios, among pregnant women, women of childbearing age, and children of various ages who consumed at least one food containing Yellow No. 6 ……………………………………………………………………………12

Figures

Figure S1. Top foods contributing to FD&C Blue No. 1 exposure estimates in children ages 0-<16 years (Typical-exposure scenario)... 14

Figure S2. Top foods contributing to FD&C Blue No. 2 exposure estimates in children ages 0-<16 years (Typical-exposure scenario)... 15

Figure S3. Top foods contributing to FD&C Green No. 3 exposure estimates in children ages 0-<16 years (Typical-exposure scenario)... 16

Figure S4. Top foods contributing to FD&C Yellow No. 5 exposure estimates in children ages 0-<16 years (Typical-exposure scenario) 17

Figure S5. Top foods contributing to FD&C Yellow No. 6 exposure estimates in children ages 0-<16 years (Typical-exposure scenario) 18

Figure S6. Children's (0-18 years) estimated total food dye intake by ethnicity (mg/kg/day).... 19

Figure S7. Women's (18-49 years) estimated total food dye intake by ethnicity (mg/kg/day)... 20

Table S1. Food dye ADI's established by the U.S FDA and JECFA.

FD&C food dye	Common Synonym	US FDA (mg/kg/day)	JECFA (WHO) (mg/kg/day)
Blue No. 1	Brilliant Blue	12.0[a]	0-6[b]
Blue No. 2	Indigo carmine, Indigotine	2.5[a]	0-5[c]
Green No. 3	Fast Green	2.5[a]	0-25[b]
Red No. 3	Erythrosine	2.5[a]	0-0.1[c]
Red No. 40	Allura Red	7.0[a]	0-7[d]
Yellow No. 5	Tartrazine	5.0[a]	0-10[d]
Yellow No. 6	Sunset Yellow	3.75[a]	0-4[e]

JECFA presents their ADIs as a range from 0 to a positive value.

Note, US FDA ADI's were approved for listed FD&C food color additives in the years 1969 to 1987 (Blue No. 1: 1969; Blue No. 2: 1987; Green No. 3: 1982; Red No. 3: 1969; Red No. 40: 1971; Yellow No. 5: 1969; and Yellow No. 6: 1986) (US FDA 2011).

Sources:

[a]US FDA. 2011. Background document for the Food Advisory Committee: Certified color additives in food and possible association with attention deficit hyperactivity disorder in children March 30-31, 2011. Food and Drug Administration / Center for Food Safety and Applied Nutrition.

[b]WHO JECFA. 2017. Evaluation of certain food additives: eighty-fourth report of the Joint FAO/WHO Expert Committee on Food Additives. (WHO Technical Report Series). 978-92-4-121016-4. World Health Organization.

[c]WHO JECFA. 2019. Evaluation of certain food additives: eighty-sixth report of the Joint FAO/WHO Expert Committee on Food Additives. (WHO Technical Report Series). 978-92-4-121023-2. World Health Organization.

[d]WHO JECFA. 2016. Evaluation of certain food additives: eighty-second report of the Joint FAO/WHO Expert Committee on Food Additives. (WHO technical report series). 978-92-4-121000-3. World Health Organization.

[e]WHO JECFA. 2011. Evaluation of certain food additives and contaminants: seventy-fourth report of the Joint FAO/WHO Expert Committee on Food Additives. (WHO technical report series). 978-92-4-120966-3. World Health Organization.

Table S2. Estimated single-day and two-day average FD&C Blue No. 1 exposure (mg/kg/day) and hazard ratios among pregnant women, women of childbearing age, and children of various ages who consumed at least one food containing Blue No. 1.

FD&C Blue No. 1			Typical-exposure scenario							High-exposure scenario						
						FDA Ratio		JECFA Ratio					FDA Ratio		JECFA Ratio	
	Total n[b]	n[c]	Mean	Median	95th%	Mean	95th%	Mean	95th%	Mean	Median	95th%	Mean	95th%	Mean	95th%
Pregnant women																
Day 1	48	44	0.01	0.006	0.04	0.001	0.003	0.001	0.003	0.02	0.01	0.10	0.002	0.008	0.002	0.008
Day 2	31	25	0.02	0.01	0.05	0.001	0.004	0.001	0.004	0.03	0.02	0.06	0.002	0.005	0.002	0.005
2-Day average[a]	42	39	0.01	0.005	0.03	0.001	0.002	0.001	0.002	0.02	0.01	0.06	0.002	0.005	0.001	0.005
Women 18-49 years																
Day 1	1048	933	0.02	0.008	0.06	0.001	0.005	0.001	0.004	0.03	0.01	0.09	0.002	0.008	0.002	0.008
Day 2	792	671	0.02	0.009	0.06	0.001	0.005	0.001	0.005	0.03	0.02	0.10	0.003	0.008	0.002	0.008
2-Day average[a]	1040	946	0.01	0.006	0.04	0.001	0.004	0.001	0.003	0.02	0.01	0.08	0.002	0.006	0.002	0.006
Children (0-<2 years)																
Day 1	177	151	0.04	0.03	0.14	0.004	0.01	0.004	0.01	0.09	0.05	0.29	0.01	0.02	0.01	0.023
Day 2	131	113	0.11	0.02	0.21	0.01	0.02	0.008	0.02	0.39	0.05	0.35	0.03	0.03	0.03	0.03
2-Day average[a]	186	163	0.05	0.02	0.12	0.004	0.01	0.004	0.01	0.16	0.04	0.22	0.01	0.02	0.01	0.02
Children (2-<5 years)																
Day 1	388	353	0.08	0.03	0.22	0.006	0.02	0.006	0.02	0.22	0.06	0.39	0.02	0.03	0.02	0.03
Day 2	300	259	0.05	0.03	0.15	0.004	0.01	0.004	0.01	0.10	0.06	0.32	0.01	0.03	0.01	0.03
2-Day average[a]	363	346	0.05	0.02	0.15	0.004	0.01	0.004	0.01	0.12	0.04	0.30	0.01	0.02	0.01	0.02
Children (5-<9 years)																
Day 1	569	536	0.05	0.03	0.13	0.004	0.01	0.004	0.01	0.09	0.06	0.23	0.01	0.02	0.01	0.02
Day 2	397	374	0.05	0.02	0.14	0.004	0.01	0.004	0.01	0.09	0.05	0.29	0.01	0.02	0.01	0.02
2-Day average[a]	501	487	0.04	0.02	0.15	0.003	0.01	0.003	0.01	0.07	0.04	0.26	0.01	0.02	0.01	0.02
Children (9-<16 years)																
Day 1	908	822	0.04	0.02	0.10	0.003	0.01	0.003	0.01	0.09	0.04	0.19	0.01	0.02	0.007	0.02
Day 2	660	598	0.03	0.01	0.11	0.003	0.009	0.003	0.008	0.06	0.03	0.18	0.01	0.01	0.005	0.01
2-Day average[a]	843	801	0.03	0.01	0.09	0.002	0.008	0.002	0.008	0.06	0.03	0.16	0.005	0.01	0.005	0.01
Youth (16-18 years)																
Day 1	342	286	0.02	0.01	0.09	0.002	0.007	0.002	0.007	0.04	0.02	0.15	0.003	0.01	0.003	0.01
Day 2	222	194	0.01	0.008	0.05	0.001	0.004	0.001	0.004	0.03	0.02	0.09	0.002	0.008	0.002	0.007
2-Day average[a]	310	288	0.01	0.007	0.05	0.001	0.004	0.001	0.004	0.02	0.01	0.09	0.002	0.007	0.002	0.007

Abbreviations: 95th%: 95th percentile; FDA: Food and Drug Administration; JECFA: Joint FAO/WHO Expert Committee on Food Additives; ADI: acceptable daily intake

Note: For FD&C Blue No. 1, the FDA ADI is 12.0 and JECFA ADI is 6 mg/kg/day.

FDA and JECFA ratios (hazard ratios)=ratios of mean and 95th percentile single-day and two-day average FD&C Blue No. 1 exposure (mg/kg/day) to FDA and JECFA ADIs

If FD&C Blue No. 1 was listed on the label for a food, but the results for that color additive were below the LOD, we assumed the AFC was present in the product at the LOD (i.e., 1.0 mg/kg).

[a]The 2-Day average estimates include individuals who completed both the Day 1 and Day 2 NHANES food consumption questionnaires.
[b]Total n=number of AFC eaters, i.e. individuals who ate at least one food containing any of the seven FD&C artificial food dyes.
[c]n=number of individuals who consumed at least one food containing FD&C Blue No. 1; means, medians, and 95th percentiles are calculated based on these individuals.

Table S3. Estimated single-day and two-day average FD&C Blue No. 2 exposure (mg/kg/day) and hazard ratios among pregnant women, women of childbearing age, and children of various ages who consumed at least one food containing Blue No. 2.

FD&C Blue No. 2			Typical-exposure scenario							High-exposure scenario						
						FDA Ratio		JECFA Ratio					FDA Ratio		JECFA Ratio	
	Total n[b]	n[c]	Mean	Median	95th%	Mean	95th%	Mean	95th%	Mean	Median	95th%	Mean	95th%	Mean	95th%
Pregnant women																
Day 1	48	23	0.008	0.002	0.05	0.003	0.02	0.002	0.01	0.02	0.004	0.15	0.008	0.06	0.004	0.03
Day 2	31	18	0.008	0.003	0.03	0.003	0.01	0.002	0.006	0.02	0.005	0.07	0.006	0.03	0.003	0.01
2-Day average[a]	42	25	0.007	0.002	0.03	0.003	0.01	0.001	0.006	0.02	0.004	0.07	0.006	0.03	0.003	0.01
Women 18-49 years																
Day 1	1048	566	0.007	0.002	0.03	0.003	0.01	0.001	0.006	0.01	0.003	0.05	0.005	0.02	0.002	0.01
Day 2	792	426	0.009	0.003	0.03	0.004	0.01	0.002	0.007	0.02	0.005	0.07	0.006	0.03	0.003	0.01
2-Day average[a]	1040	645	0.005	0.002	0.02	0.002	0.01	0.001	0.004	0.009	0.003	0.04	0.004	0.01	0.002	0.01
Children (0-<2 years)																
Day 1	177	91	0.05	0.01	0.24	0.02	0.09	0.01	0.05	0.06	0.01	0.34	0.03	0.13	0.01	0.07
Day 2	131	68	0.04	0.009	0.20	0.02	0.08	0.008	0.04	0.09	0.01	0.24	0.04	0.10	0.02	0.05
2-Day average[a]	186	108	0.03	0.007	0.11	0.01	0.05	0.006	0.02	0.05	0.009	0.24	0.02	0.09	0.010	0.05
Children (2-<5 years)																
Day 1	388	227	0.02	0.005	0.08	0.01	0.03	0.005	0.02	0.05	0.005	0.20	0.02	0.08	0.01	0.04
Day 2	300	155	0.03	0.009	0.14	0.01	0.05	0.007	0.03	0.06	0.01	0.21	0.02	0.08	0.01	0.04
2-Day average[a]	363	235	0.02	0.006	0.08	0.008	0.03	0.004	0.02	0.04	0.007	0.13	0.01	0.05	0.01	0.03
Children (5-<9 years)																
Day 1	569	341	0.02	0.006	0.09	0.009	0.03	0.004	0.02	0.04	0.007	0.21	0.02	0.08	0.01	0.04
Day 2	397	232	0.03	0.007	0.08	0.01	0.03	0.005	0.02	0.05	0.009	0.24	0.02	0.09	0.01	0.05
2-Day average[a]	501	360	0.02	0.007	0.06	0.007	0.03	0.003	0.01	0.03	0.01	0.13	0.01	0.05	0.01	0.03
Children (9-<16 years)																
Day 1	908	500	0.02	0.005	0.06	0.009	0.02	0.005	0.01	0.04	0.007	0.14	0.02	0.06	0.008	0.03
Day 2	660	348	0.02	0.009	0.08	0.009	0.03	0.005	0.02	0.04	0.01	0.14	0.02	0.06	0.008	0.03
2-Day average[a]	843	570	0.02	0.006	0.05	0.006	0.02	0.003	0.01	0.03	0.008	0.09	0.01	0.03	0.005	0.02
Youth (16-18 years)																
Day 1	342	159	0.01	0.002	0.04	0.005	0.02	0.002	0.01	0.02	0.004	0.09	0.01	0.04	0.004	0.02
Day 2	222	110	0.01	0.004	0.04	0.004	0.02	0.002	0.01	0.02	0.005	0.09	0.01	0.04	0.004	0.02
2-Day average[a]	310	187	0.007	0.002	0.03	0.003	0.01	0.001	0.006	0.01	0.003	0.06	0.004	0.02	0.002	0.01

Abbreviations: 95th%: 95th percentile; FDA: Food and Drug Administration; JECFA: Joint FAO/WHO Expert Committee on Food Additives; ADI: acceptable daily intake

Note: Note: For FD&C Blue No. 2, the FDA ADI is 2.5 and JECFA ADI is 5 mg/kg/day.

FDA and JECFA ratios (hazard ratios)=ratios of mean and 95th percentile single-day and two-day average FD&C Blue No. 2 exposure (mg/kg/day) to FDA and JECFA ADIs

If FD&C Blue No. 2 was listed on the label for a food, but the results for that color additive were below the LOD, we assumed the AFC was present in the product at the LOD (i.e., 1.0 mg/kg).

[a]The 2-Day average estimates include individuals who completed both the Day 1 and Day 2 NHANES food consumption questionnaires.
[b]Total n=number of AFC eaters, i.e. individuals who ate at least one food containing any of the seven FD&C artificial food dyes.
[c]n=number of individuals who consumed at least one food containing FD&C Blue No. 2; means, medians, and 95th percentiles are calculated based on these individuals.

Table S4. Estimated single-day and two-day average FD&C Green No. 3 exposure (mg/kg/day) and hazard ratios among pregnant women, women of childbearing age, and children of various ages who consumed at least one food containing Green No. 3.

FD&C Green No. 3			Typical-exposure scenario							High-exposure scenario						
						FDA Ratio		JECFA Ratio					FDA Ratio		JECFA Ratio	
	Total n[b]	n[c]	Mean	Median	95th%	Mean	95th%	Mean	95th%	Mean	Median	95th%	Mean	95th%	Mean	95th%
Pregnant women																
Day 1	48	3	0.002	0.002	0.002	0.001	0.001	0.0001	0.0001	0.002	0.002	0.002	0.0008	0.001	0.0001	0.0001
Day 2	31	2	0.003	0.003	0.004	0.001	0.002	0.0001	0.0002	0.003	0.003	0.004	0.001	0.002	0.0001	0.0002
2 -Day average[a]	42	4	0.001	0.001	0.003	0.0004	0.001	0.00004	0.0001	0.001	0.001	0.003	0.0004	0.001	0.00004	0.0001
Women 18-49 years																
Day 1	1048	102	0.002	0.001	0.004	0.0008	0.002	0.00008	0.0002	0.002	0.001	0.004	0.0008	0.002	0.00008	0.0002
Day 2	792	73	0.002	0.002	0.005	0.001	0.002	0.0001	0.0002	0.002	0.002	0.005	0.001	0.002	0.0001	0.0002
2 -Day average[a]	1040	145	0.001	0.001	0.003	0.0004	0.001	0.00004	0.0001	0.001	0.001	0.003	0.0004	0.001	0.00004	0.0001
Children (0-<2 years)																
Day 1	177	13	0.003	0.004	0.007	0.001	0.003	0.0001	0.0003	0.003	0.004	0.007	0.001	0.003	0.0001	0.0003
Day 2	131	9	0.005	0.003	0.009	0.002	0.004	0.0002	0.0004	0.005	0.003	0.009	0.002	0.004	0.0002	0.0004
2-Day average[a]	186	17	0.002	0.002	0.004	0.001	0.002	0.0001	0.0002	0.002	0.002	0.004	0.001	0.002	0.0001	0.0002
Children (2-<5 years)																
Day 1	388	32	0.005	0.005	0.01	0.002	0.004	0.0002	0.0004	0.005	0.005	0.010	0.002	0.004	0.0002	0.0004
Day 2	300	25	0.005	0.004	0.012	0.002	0.005	0.0002	0.0005	0.005	0.004	0.010	0.002	0.005	0.0002	0.0005
2-Day average[a]	363	42	0.003	0.002	0.006	0.001	0.002	0.0001	0.0002	0.003	0.002	0.006	0.001	0.002	0.0001	0.0002
Children (5-<9 years)																
Day 1	569	69	0.004	0.003	0.01	0.002	0.004	0.0002	0.0004	0.004	0.003	0.010	0.002	0.004	0.0002	0.0004
Day 2	397	52	0.004	0.003	0.008	0.002	0.003	0.0002	0.0003	0.004	0.003	0.008	0.002	0.003	0.0002	0.0003
2-Day average[a]	501	89	0.002	0.002	0.005	0.001	0.002	0.0001	0.0002	0.002	0.002	0.005	0.001	0.002	0.0001	0.0002
Children (9-<16 years)																
Day 1	908	103	0.003	0.002	0.008	0.001	0.003	0.0001	0.0003	0.003	0.002	0.008	0.001	0.003	0.0001	0.0003
Day 2	660	76	0.004	0.003	0.008	0.002	0.003	0.0002	0.0003	0.004	0.003	0.008	0.002	0.003	0.0002	0.0003
2-Day average[a]	843	144	0.002	0.002	0.004	0.0008	0.002	0.00008	0.0002	0.002	0.002	0.004	0.0008	0.002	0.00008	0.0002
Youth (16-18 years)																
Day 1	342	20	0.002	0.002	0.004	0.001	0.002	0.0001	0.0002	0.002	0.002	0.004	0.001	0.002	0.0001	0.0002
Day 2	222	13	0.003	0.002	0.009	0.001	0.004	0.0001	0.0004	0.003	0.002	0.009	0.001	0.004	0.0001	0.0004
2-Day average[a]	310	29	0.001	0.001	0.004	0.0004	0.002	0.00004	0.0002	0.001	0.001	0.004	0.0004	0.002	0.00004	0.0002

Abbreviations: 95th%: 95th percentile; FDA: Food and Drug Administration; JECFA: Joint FAO/WHO Expert Committee on Food Additives; ADI: acceptable daily intake

Note: For FD&C Green No. 3 the FDA ADI is 2.5 and JECFA ADI is 25 mg/kg/day.

FDA and JECFA ratios (hazard ratios)=ratios of mean and 95th percentile single-day and two-day average FD&C Green No. 3 exposure (mg/kg/day) to FDA and JECFA ADIs.

If FD&C Green No. 3 was listed on the label for a food, but the results for that color additive were below the LOD, we assumed the AFC was present in the product at the LOD (i.e., 1.0 mg/kg).

[a]The 2-Day average estimates include individuals who completed both the Day 1 and Day 2 NHANES food consumption questionnaires.
[b]Total n=number of AFC eaters, i.e., individuals who ate at least one food containing any of the seven FD&C artificial food dyes.
[c]n=number of individuals who consumed at least one food containing Green No. 3 means, medians, and 95th percentiles are calculated based on these individuals.

Table S5. Estimated single-day and two-day average FD&C Yellow No. 5 exposure (mg/kg/day) and hazard ratios among pregnant women, women of childbearing age, and children of various ages who consumed at least one food containing Yellow No. 5.

FD&C Yellow No. 5			Typical-exposure scenario							High-exposure scenario						
						FDA Ratio		JECFA Ratio					FDA Ratio		JECFA Ratio	
	Total n[b]	n[c]	Mean	Median	95th%	Mean	95th%	Mean	95th%	Mean	Median	95th%	Mean	95th%	Mean	95th%
Pregnant women																
Day 1	48	42	0.05	0.02	0.15	0.01	0.03	0.005	0.02	0.07	0.04	0.23	0.01	0.05	0.01	0.02
Day 2	31	29	0.03	0.02	0.05	0.005	0.01	0.003	0.01	0.05	0.02	0.21	0.01	0.04	0.005	0.02
2 -Day average[a]	42	36	0.03	0.02	0.09	0.006	0.02	0.003	0.01	0.05	0.03	0.14	0.01	0.03	0.01	0.01
Women 18-49 years																
Day 1	1048	947	0.05	0.02	0.16	0.01	0.03	0.005	0.02	0.08	0.04	0.29	0.02	0.06	0.01	0.03
Day 2	792	715	0.05	0.02	0.18	0.01	0.04	0.005	0.02	0.08	0.03	0.28	0.02	0.06	0.01	0.03
2 -Day average[a]	1040	973	0.03	0.02	0.13	0.01	0.03	0.003	0.01	0.06	0.03	0.20	0.01	0.04	0.01	0.02
Children (0-<2 years)																
Day 1	177	169	0.13	0.07	0.42	0.03	0.08	0.01	0.04	0.22	0.12	0.78	0.04	0.16	0.02	0.08
Day 2	131	121	0.19	0.08	0.76	0.04	0.15	0.02	0.08	0.27	0.14	1.02	0.05	0.20	0.03	0.10
2-Day average[a]	186	176	0.12	0.06	0.45	0.02	0.090	0.01	0.04	0.19	0.10	0.76	0.04	0.15	0.02	0.08
Children (2-<5 years)																
Day 1	388	375	0.14	0.06	0.56	0.03	0.11	0.01	0.06	0.23	0.11	0.80	0.05	0.16	0.02	0.08
Day 2	300	286	0.16	0.09	0.58	0.03	0.12	0.02	0.06	0.25	0.13	0.85	0.05	0.17	0.03	0.08
2-Day average[a]	363	353	0.12	0.07	0.39	0.02	0.08	0.01	0.04	0.19	0.11	0.51	0.04	0.10	0.02	0.05
Children (5-<9 years)																
Day 1	569	548	0.11	0.06	0.38	0.02	0.08	0.01	0.04	0.18	0.10	0.66	0.04	0.13	0.02	0.07
Day 2	397	381	0.11	0.05	0.46	0.02	0.09	0.01	0.05	0.18	0.08	0.60	0.04	0.12	0.02	0.06
2-Day average[a]	501	495	0.09	0.05	0.31	0.02	0.06	0.01	0.03	0.15	0.09	0.48	0.03	0.10	0.02	0.05
Children (9-<16 years)																
Day 1	908	846	0.09	0.04	0.35	0.02	0.07	0.009	0.04	0.17	0.06	0.65	0.03	0.13	0.02	0.07
Day 2	660	627	0.08	0.04	0.33	0.02	0.07	0.008	0.03	0.14	0.06	0.56	0.03	0.11	0.01	0.06
2-Day average[a]	843	816	0.07	0.04	0.26	0.01	0.05	0.007	0.03	0.12	0.06	0.40	0.02	0.08	0.01	0.04
Youth (16-18 years)																
Day 1	342	302	0.06	0.03	0.20	0.01	0.04	0.006	0.02	0.10	0.04	0.34	0.02	0.07	0.01	0.03
Day 2	222	206	0.03	0.03	0.17	0.01	0.03	0.005	0.02	0.08	0.04	0.26	0.02	0.05	0.01	0.03
2-Day average[a]	310	294	0.02	0.02	0.12	0.01	0.02	0.004	0.01	0.07	0.04	0.27	0.01	0.05	0.01	0.03

Abbreviations: 95th%: 95th percentile; FDA: Food and Drug Administration; JECFA: Joint FAO/WHO Expert Committee on Food Additives; ADI: acceptable daily intake

Note: For FD&C Yellow No. 5, the FDA ADI is 5.0 and JECFA ADI is 10 mg/kg/day.

FDA and JECFA ratios (hazard ratios)=ratios of mean and 95th percentile single-day and two-day average FD&C Yellow No. 5 exposure (mg/kg/day) to FDA and JECFA ADIs.

If FD&C Yellow No 5 was listed on the label for a food, but the results for that color additive were below the LOD, we assumed the AFC was present in the product at the LOD (i.e., 1.0 mg/kg).

[a]The 2-Day average estimates include individuals who completed both the Day 1 and Day 2 NHANES food consumption questionnaires.
[b]Total n=number of AFC eaters, i.e., individuals who ate at least one food containing any of the seven FD&C artificial food dyes.
[c]n=number of individuals who consumed at least one food containing Yellow No. 5; means, medians, and 95th percentiles are calculated based on these individuals.

Table S6. Estimated single-day and two-day average FD&C Yellow No. 6 exposure (mg/kg/day) and hazard ratios among pregnant women, women of childbearing age, and children of various ages who consumed at least one food containing Yellow No. 6.

FD&C Yellow No. 6			Typical-exposure scenario							High-exposure scenario						
						FDA Ratio		JECFA Ratio					FDA Ratio		JECFA Ratio	
	Total n[b]	n[c]	Mean	Median	95th%	Mean	95th%	Mean	95th%	Mean	Median	95th%	Mean	95th%	Mean	95th%
Pregnant women																
Day 1	48	39	0.05	0.01	0.18	0.01	0.05	0.01	0.05	0.07	0.02	0.30	0.02	0.08	0.02	0.08
Day 2	31	27	0.03	0.008	0.12	0.01	0.03	0.01	0.03	0.04	0.01	0.20	0.01	0.05	0.01	0.05
2-Day average[a]	42	37	0.03	0.01	0.12	0.01	0.03	0.01	0.03	0.05	0.01	0.21	0.01	0.06	0.01	0.05
Women 18-49 years																
Day 1	1048	898	0.06	0.01	0.29	0.02	0.08	0.02	0.07	0.08	0.02	0.37	0.02	0.10	0.02	0.09
Day 2	792	692	0.05	0.01	0.20	0.01	0.05	0.01	0.05	0.07	0.01	0.28	0.02	0.08	0.02	0.07
2-Day average[a]	1040	933	0.04	0.01	0.18	0.01	0.05	0.01	0.04	0.06	0.02	0.26	0.02	0.07	0.01	0.06
Children (0-<2 years)																
Day 1	177	160	0.14	0.06	0.50	0.04	0.13	0.03	0.13	0.22	0.08	0.87	0.06	0.23	0.05	0.22
Day 2	131	120	0.20	0.08	0.72	0.05	0.19	0.05	0.18	0.47	0.10	0.94	0.13	0.25	0.12	0.24
2-Day average[a]	186	170	0.13	0.07	0.42	0.03	0.11	0.03	0.11	0.26	0.09	0.75	0.07	0.20	0.06	0.19
Children (2-<5 years)																
Day 1	388	359	0.18	0.06	0.84	0.05	0.22	0.05	0.21	0.35	0.09	1.10	0.09	0.29	0.09	0.27
Day 2	300	276	0.16	0.08	0.54	0.04	0.14	0.04	0.14	0.23	0.10	0.86	0.06	0.23	0.06	0.22
2-Day average[a]	363	353	0.14	0.07	0.48	0.04	0.13	0.03	0.12	0.22	0.10	0.69	0.06	0.18	0.06	0.17
Children (5-<9 years)																
Day 1	569	539	0.15	0.08	0.51	0.04	0.13	0.04	0.13	0.23	0.11	0.84	0.06	0.22	0.06	0.21
Day 2	397	376	0.11	0.05	0.47	0.03	0.13	0.03	0.12	0.16	0.07	0.64	0.04	0.17	0.04	0.16
2-Day average[a]	501	493	0.11	0.06	0.37	0.03	0.10	0.03	0.09	0.16	0.09	0.60	0.04	0.16	0.04	0.15
Children (9-<16 years)																
Day 1	908	837	0.11	0.05	0.36	0.03	0.10	0.03	0.09	0.18	0.07	0.56	0.05	0.15	0.04	0.14
Day 2	660	619	0.09	0.03	0.35	0.02	0.09	0.02	0.09	0.13	0.05	0.44	0.03	0.12	0.03	0.11
2-Day average[a]	843	816	0.08	0.05	0.28	0.02	0.07	0.02	0.07	0.12	0.07	0.37	0.03	0.10	0.03	0.09
Youth (16-18 years)																
Day 1	342	299	0.07	0.02	0.26	0.02	0.07	0.02	0.06	0.10	0.03	0.39	0.03	0.10	0.02	0.10
Day 2	222	202	0.05	0.02	0.22	0.01	0.06	0.01	0.06	0.08	0.02	0.32	0.02	0.08	0.02	0.08
2-Day average[a]	310	291	0.04	0.02	0.17	0.01	0.04	0.01	0.04	0.06	0.02	0.26	0.02	0.07	0.02	0.07

Abbreviations: 95th%: 95th percentile; FDA: Food and Drug Administration; JECFA: Joint FAO/WHO Expert Committee on Food Additives; ADI: acceptable daily intake

Note: For FD&C Yellow No. 6, the FDA ADI is 3.75 and JECFA ADI is 4 mg/kg/day.

FDA and JECFA ratios (hazard ratios)=ratios of mean and 95th percentile single-day and two-day average FD&C Yellow No. 6 exposure (mg/kg/day) to FDA and JECFA ADIs.

If FD&C Yellow No. 6 was listed on the label for a food, but the results for that color additive were below the LOD, we assumed the AFC was present in the product at the LOD (i.e., 1.0 mg/kg).

[a]The 2-Day average estimates include individuals who completed both the Day 1 and Day 2 NHANES food consumption questionnaires.
[b]Total n=number of AFC eaters, i.e. individuals who ate at least one food containing any of the seven FD&C artificial food dyes.
[c]n=number of individuals who consumed at least one food containing Yellow No. 6; means, medians, and 95th percentiles are calculated based on these individuals.

Figure S1. Top foods contributing to FD&C Blue No. 1 exposure estimates in children ages 0-<16 years (Typical-exposure scenario) who consumed at least one food containing Blue No. 1.

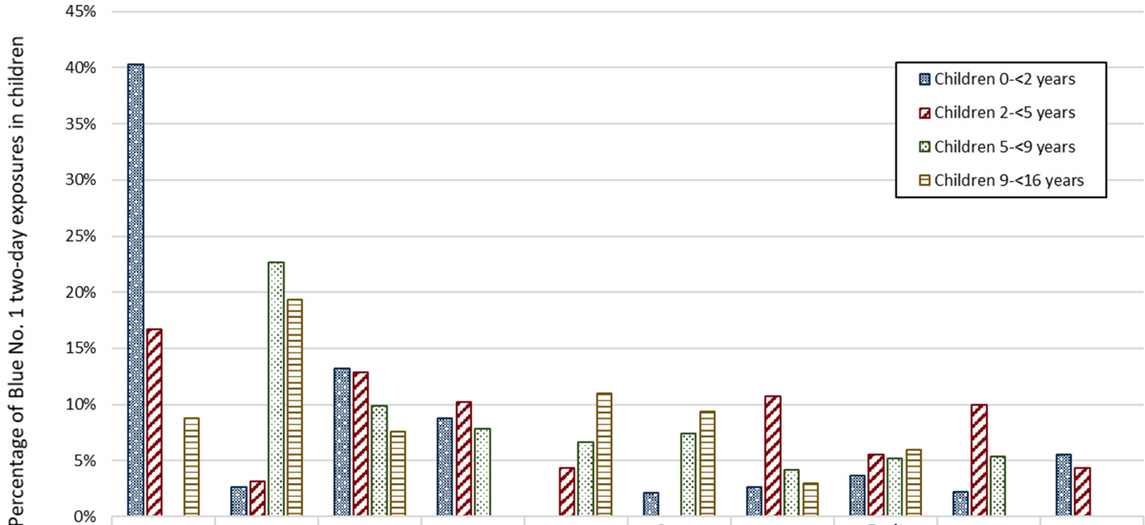

	Frostings and icings	Ice cream cones	Fruit juice drink	Fruit leather and fruit snack	Soft drinks	Sugar sprinkles/ baking decoration	Breakfast cereal	Fruit flavored drink, powdered	Ice pop	Yogurt
Children 0-<2 years	40%	3%	13%	9%	0%	2%	3%	4%	2%	6%
Children 2-<5 years	17%	3%	13%	10%	4%	0%	11%	6%	10%	4%
Children 5-<9 years	0%	23%	10%	8%	7%	7%	4%	5%	5%	0%
Children 9-<16 years	9%	19%	8%	0%	11%	9%	3%	6%	0%	0%

Figure S2. Top foods contributing to FD&C Blue No. 2 exposure estimates in children ages 0-<16 years (Typical-exposure scenario) who consumed at least one food containing Blue No. 2

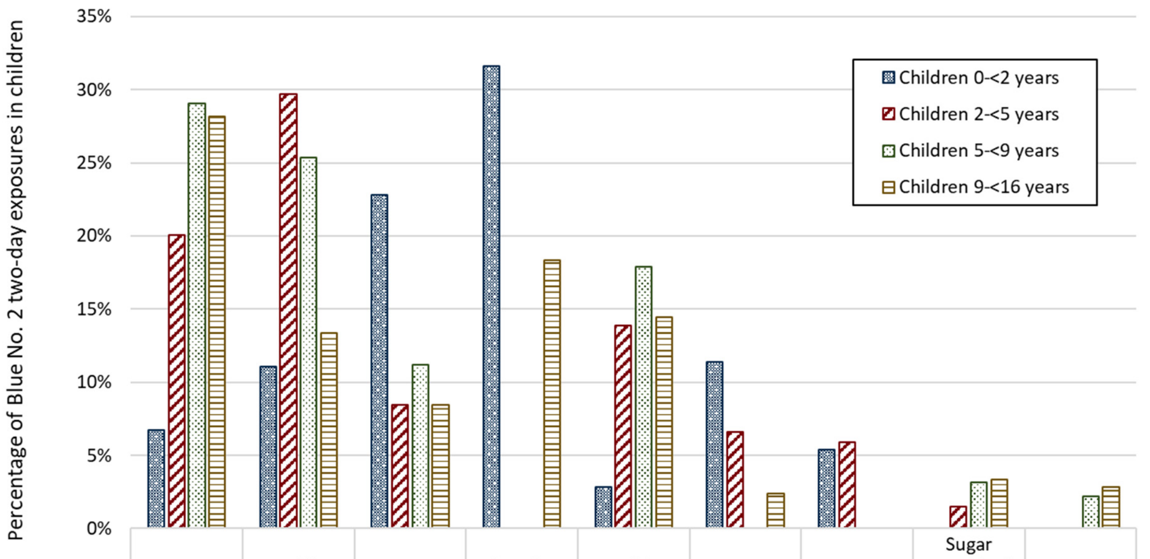

	Ice cream	Breakfast cereal	Fruit muffin	Chocolate pudding	Breakfast tart	Frostings and icings	Yogurt	Sugar sprinkles/ baking decoration	Fondant
Children 0-<2 years	7%	11%	23%	32%	3%	11%	5%	0%	0%
Children 2-<5 years	20%	30%	8%	0%	14%	7%	6%	1%	0%
Children 5-<9 years	29%	25%	11%	0%	18%	0%	0%	3%	2%
Children 9-<16 years	28%	13%	8%	18%	14%	2%	0%	3%	3%

Figure S3. Top foods contributing to FD&C Green No. 3 exposure estimates in children ages 0-<16 years (Typical-exposure scenario) who consumed at least one food containing Green No. 3.

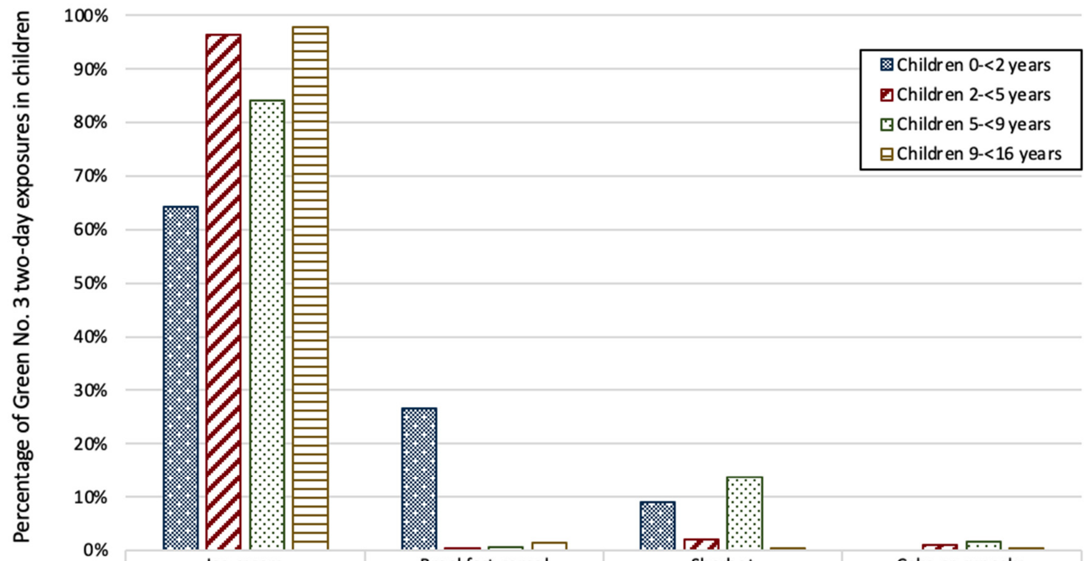

	Ice cream	Breakfast cereal	Sherbet	Cake or cupcake
Children 0-<2 years	64%	27%	9%	0%
Children 2-<5 years	96%	0%	2%	1%
Children 5-<9 years	84%	1%	14%	2%
Children 9-<16 years	98%	1%	0%	0%

Figure S4. Top foods contributing to FD&C Yellow No. 5 exposure estimates in children ages 0-<16 years (Typical-exposure scenario) who consumed at least one food containing Yellow No. 5.

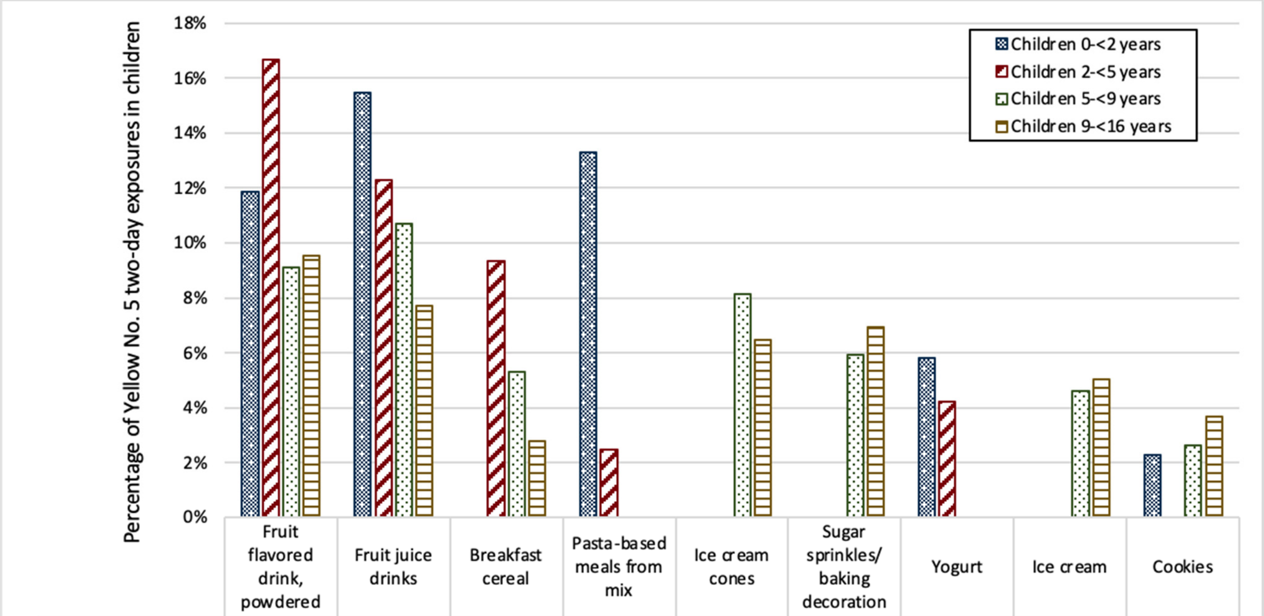

	Fruit flavored drink, powdered	Fruit juice drinks	Breakfast cereal	Pasta-based meals from mix	Ice cream cones	Sugar sprinkles/ baking decoration	Yogurt	Ice cream	Cookies
Children 0-<2 years	12%	15%	0%	13%	0%	0%	6%	0%	2%
Children 2-<5 years	17%	12%	9%	2%	0%	0%	4%	0%	0%
Children 5-<9 years	9%	11%	5%	0%	8%	6%	0%	5%	3%
Children 9-<16 years	10%	8%	3%	0%	6%	7%	0%	5%	4%

Figure S5. Top foods contributing to FD&C Yellow No. 6 exposure estimates in children ages 0-<16 years (Typical-exposure scenario) who consumed at least one food containing Yellow No. 6.

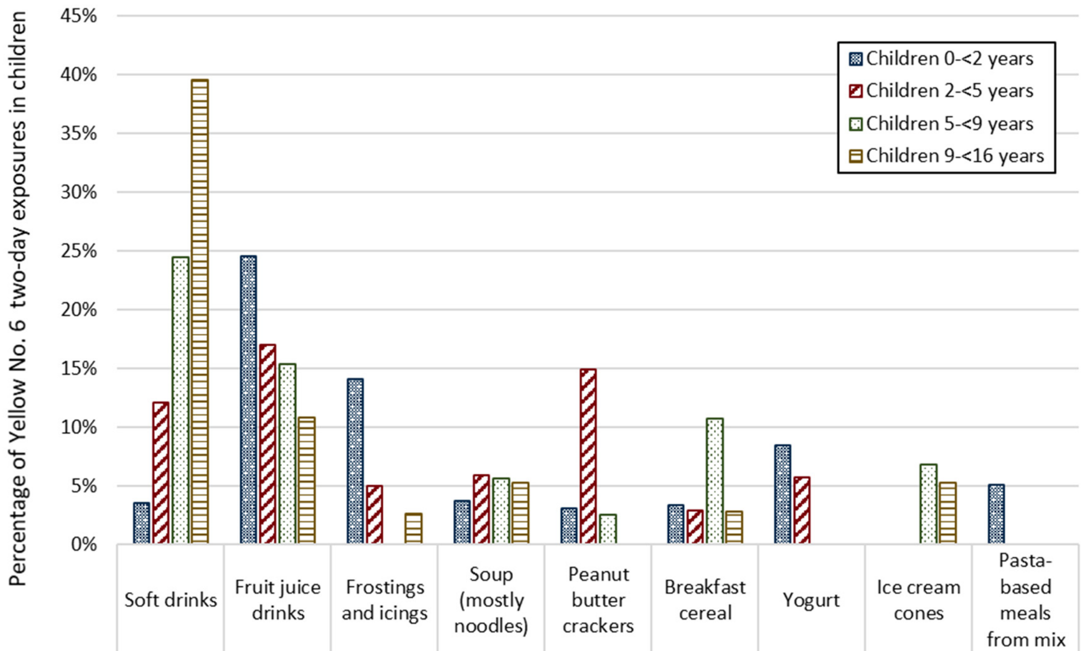

	Soft drinks	Fruit juice drinks	Frostings and icings	Soup (mostly noodles)	Peanut butter crackers	Breakfast cereal	Yogurt	Ice cream cones	Pasta-based meals from mix
Children 0-<2 years	4%	25%	14%	4%	3%	3%	9%	0%	5%
Children 2-<5 years	12%	17%	5%	6%	15%	3%	6%	0%	0%
Children 5-<9 years	24%	15%	0%	6%	3%	11%	0%	7%	0%
Children 9-<16 years	40%	11%	3%	5%	0%	3%	0%	5%	0%

Figure S6. Cumulative distribution of children's (0-18 years) two-day average total food dye intake estimates (natural log-transformed mg/kg/day) by ethnicity among "eaters" of at least one food containing an artificial food color.

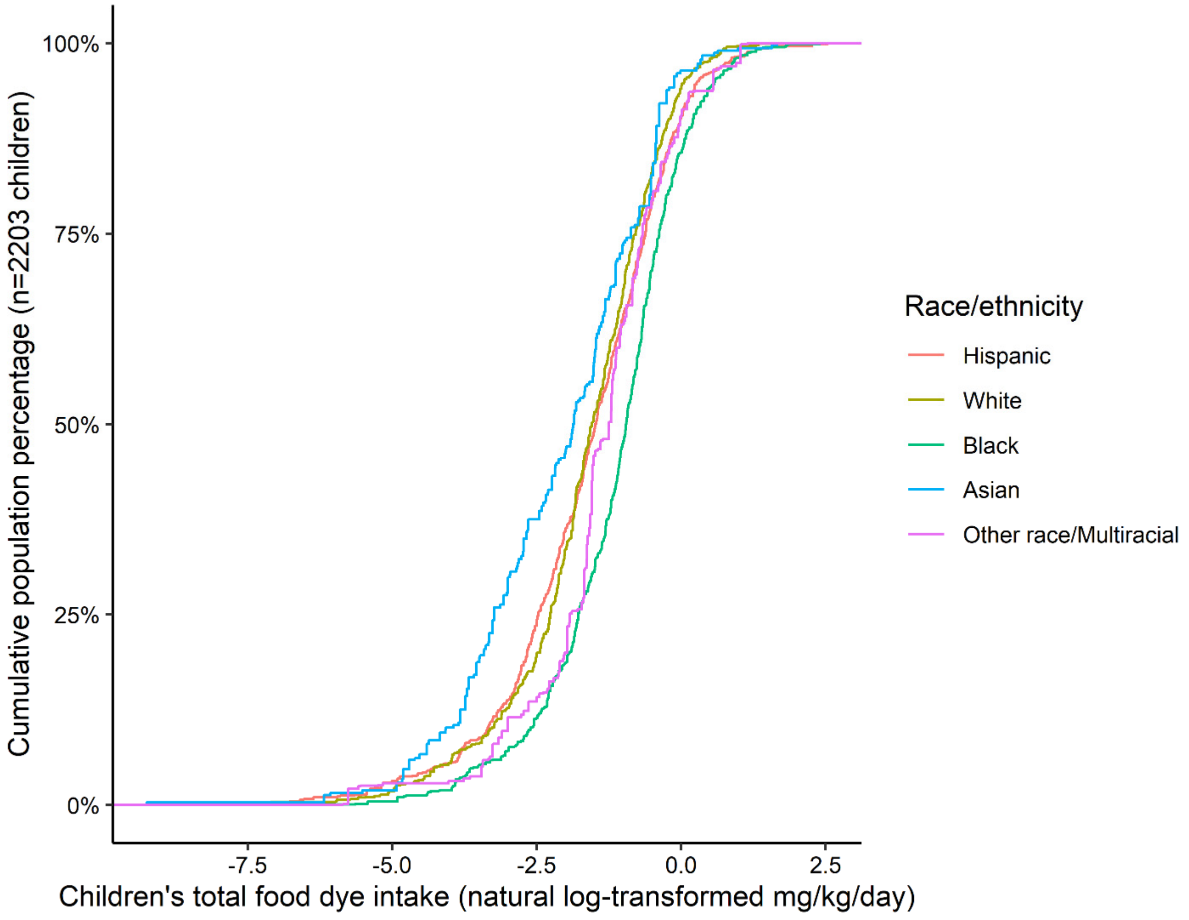

Race/ethnicity: Hispanic (Mexican American/Other Hispanic); White (non-Hispanic White); Black (Non-Hispanic Black); Asian (non-Hispanic Asian); and Other race/Multiracial.

Figure S7. Cumulative distribution of women's (18-49 years) two-day average total food dye intake estimates (natural log-transformed mg/kg/day) by ethnicity among "eaters" of at least one food containing an artificial food color.

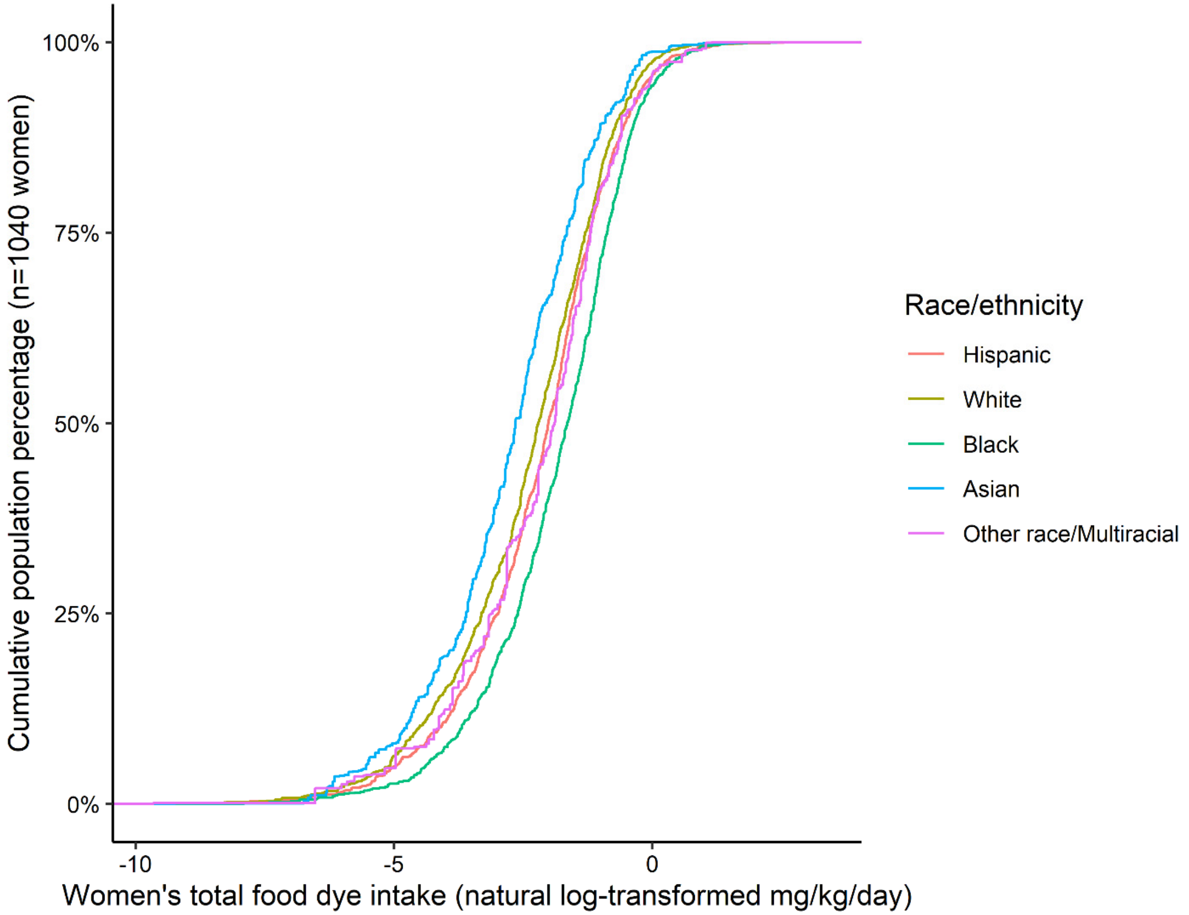

Race/ethnicity: Hispanic (Mexican American/Other Hispanic); White (non-Hispanic White); Black (Non-Hispanic Black); Asian (non-Hispanic Asian); and Other race/Multiracial.
